# Hagfish genome reveals parallel evolution of 7SL RNA-derived SINEs

**DOI:** 10.1186/s13100-020-00210-2

**Published:** 2020-05-22

**Authors:** Kenji K. Kojima

**Affiliations:** Genetic Information Research Institute, Cupertino, CA 95014 USA

## Abstract

**Background:**

Short interspersed elements (SINEs) are ubiquitous components of eukaryotic genomes. SINEs are composite transposable elements that are mobilized by non-long terminal repeat (non-LTR) retrotransposons, also called long interspersed elements (LINEs). The 3′ part of SINEs usually originated from that of counterpart non-LTR retrotransposons. The 5′ part of SINEs mostly originated from small RNA genes. SINE1 is a group of SINEs whose 5′ part originated from 7SL RNA, and is represented by primate *Alu* and murine *B1*. Well-defined SINE1 has been found only from Euarchontoglires, a group of mammals, in contrast to the wide distribution of SINE2, which has a tRNA-derived sequence, from animals to plants to protists. Both *Alu* and *B1* are mobilized by *L1*-type non-LTR retrotransposons, which are the only lineage of autonomous non-LTR retrotransposons active in these mammalian lineages.

**Results:**

Here a new lineage of SINE1 is characterized from the seashore hagfish *Eptatretus burgeri* genome. This SINE1 family, designated *SINE1*-*1_EBu*, is young, and is transposed by *RTE*-type non-LTR retrotransposon, not *L1*-type. Comparison with other SINE families from hagfish indicated the birth of *SINE1*-*1_EBu* through chimera formation of a 7SL RNA-derived sequence and an older tRNA-derived SINE family. It reveals parallel evolution of SINE1 in two vertebrate lineages with different autonomous non-LTR retrotransposon partners. The comparison between two SINE1 lineages supports that the RNA secondary structure of the *Alu* domain of 7SL RNA is required for the efficient retrotransposition.

**Conclusions:**

The hagfish SINE1 is the first evident SINE1 family found outside of Euarchontoglires. Independent evolution of SINE1 with similar RNA secondary structure originated in 7SL RNA indicates the functional importance of 7SL RNA-derived sequence in the proliferation of SINEs.

## Introduction

Short interspersed elements (SINEs) are composite mobile elements that can mobilize dependent on the help of counterpart long interspersed elements (LINEs), also called non-long terminal repeat (non-LTR) retrotransposons [1, 2]. SINEs are composed of several independently-originated regions, called head, body, and tail. These three regions are not always present in all SINEs, and sometimes, more than one independently derived sequences constitute one of these regions.

The heads of SINEs typically originated from non-coding RNAs such as 7SL RNA, tRNA, 5S rRNA or small nuclear RNA (snRNA) [3, 4]. The heads serve primarily as internal promoters for the efficient transcription by RNA polymerase III [5]. SINEs with tRNA-originated heads, called SINE2, are the most common SINEs and widely distributed among eukaryotes [6]. SINEs with 5S rRNA-derived heads, called SINE3 are the second widely distributed and have been found from various vertebrates [3, 6]. SINEs with 7SL RNA-derived heads are called SINE1. Well-defined examples of SINE1 are only found in one lineage of mammals, Euarchontoglires, which is composed of 5 orders: primates, flying lemurs, tree shrews, rodents, and lagomorphs [7]. Two of the best studied SINE families, *Alu* from humans and *B1* from mice, belong to SINE1. A putative SINE1 family was also found from marsupials [8], but this family, designated as *P7SL_MD* in Repbase, is composed of a full-length 7SL RNA and a 3’ poly A tail. It is structurally indistinguishable from retrocopies of 7SL RNAs. *P7SL_MD* was multiplicated into over 12,000 copies in the common ancestor of marsupials [9]. An expansion of putative retrocopies of 7SL RNA is also reported from the guinea pig genome [10].

*Alu* is the abundant repetitive sequence accounting for 11% of the human genome [11]. A typical *Alu* sequence is a dimer composed by two 7SL RNA-derived monomer units connected by an A-rich linker [12]. Dimeric *Alu* elements are considered to have been originally generated through a fusion between free left *Alu* monomer (*FLAM*)-*C* and free right *Alu* monomer (*FRAM*) [13]. Two other types of *Alu* monomers, fossil *Alu* monomer (*FAM*) and *FLAM*-*A* likely predated *FLAM*-*C* and *FRAM* [14, 15]. *FLAM*-*A* is nearly identical to the rodent SINE family *PB1* [16], and thus, *FLAM*-*A/PB1* was likely born in the common ancestor of Euarchontoglires [7, 17]. Most rodents have families of monomeric 7SL RNA-derived SINEs called *B1* [18]. Tree shrews have Tu type SINEs, which show chimeric structures between 7SL RNA-derived SINEs and tRNA-derived SINEs [17]. SINE families originating by the fusion of tRNA-derived SINEs and 7SL RNA-derived SINEs were also found in the bushbaby *Otolemur garnettii*, designated as *GarnAlu* [19]. Active monomeric 7SL RNA-derived SINE families (*Platy*-*1*) were also found from the common marmoset *Callithrix jacchus* [20]. All SINE1 families found in Euarchontoglires are considered to be descendants of the common ancestor.

*Alu* and *B1* are mobilized by the two proteins, L1ORF1p and L1ORF2p, encoded by *L1* [2, 21]. *L1* is distributed widely in eukaryotes [6]. Many mammals including humans and mice retain young *L1* lineages which are active or have been active recently. Unlike other non-LTR retrotransposons, mammalian *L1* does not require the conserved RNA secondary structure in the 3′ UTR for the recognition of the template RNA for reverse transcription [22]. Due to this relaxed recognition, *Alu* and *B1* RNAs as well as any polyadenylated mRNAs, can be mobilized by the *L1* machinery. Both *Alu* and *B1* are composed solely by the sequences of 7SL RNA. The relaxed recognition by the *L1* machinery is also seen in plants, but it is considered that the machineries of non-mammalian *L1* as well as of other non-LTR retrotransposons recognize the RNA secondary structure besides the 3’-polyA tail [1, 23].

SINE1 families from Euarchontoglires share the sequence corresponding to the regions 1-63, 76-83, and 267-299 of the human 7SL RNA [7]. 7SL RNA-derived heads in SINE1 families from Euarchontoglires retain two functions. The internal promoter composed by two boxes (A box and B box), which is essential for the transcription of 7SL RNA, is located at 6-15 (A box) and at 76-86 (B box) in the human 7SL RNA [5]. 7SL RNA is a component of the signal recognition particle (SRP), which interacts with the ribosome. SINE1 families lack the central S domain of 7SL RNA, but retain the *Alu* domain composed by the 5′ and 3′ regions of 7SL RNA. The binding of *Alu* domain with SRP9/14 is required for the retrotransposition [24]. The binding of *Alu* RNA with SRP9/14 is proposed to be the mechanism of the efficient *trans*-mobilization by the *L1* machinery.

Here, a new SINE1 lineage is characterized from the seashore hagfish *Eptatretus burgeri* genome. This SINE1 family, designated *SINE1*-*1_EBu*, is young, and seems transposed by an *RTE*-type non-LTR retrotransposon family, not *L1*-type. It reveals parallel evolution of SINE1 in two vertebrate lineages with different autonomous non-LTR retrotransposon partners. The comparison between two SINE1 lineages indicates that the RNA secondary structure of the *Alu* domain is required for the efficient retrotransposition.

## Results

### Identification of a novel SINE1 family from the seashore hagfish genome

**Fig. 1:**
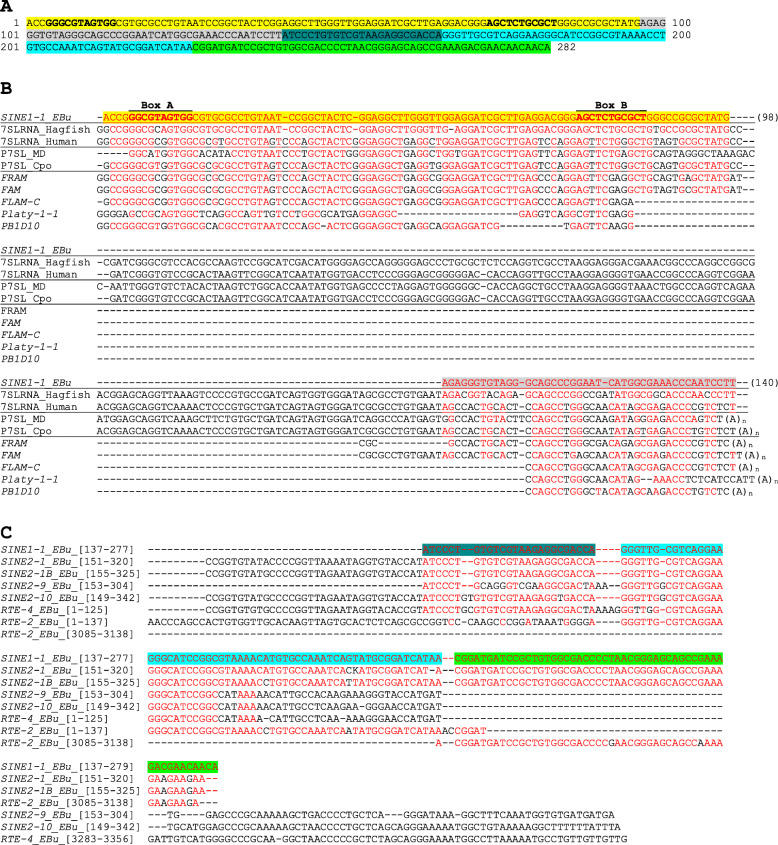
Structure of *SINE1*-*1_EBu*. Segments showing similarity to different repeats are highlighted in different colors. Box A and Box B of RNA polymerase III promoter are in boldface. Nucleotides identical to *SINE1*-*1_EBu* consensus in alignments are colored in red. **a** Full-length sequence of *SINE1*-*1_EBu*. **b** Sequence alignment between *SINE1*-*1_EBu*, hagfish and human 7SL RNA genes and 7SL RNA-derived SINEs from Euarchontoglires. One 7SL RNA gene from hagfish in the accession number FYBX02009602 is used for alignment. *FRAM*, *FAM* and *FLAM-C* are ancestral primate SINE1 families, and they are monomers. *Platy*-*1*-*1* is a family from the common marmoset. *PB1D10* is an ancestral rodent SINE family. **c** Sequence alignment between hagfish SINEs related to *SINE1*-*1_EBu* and their putative autonomous counterparts. Positions inside of entire consensus sequences are shown in parentheses

During the repeat analysis of the seashore hagfish genome, a repeat family was identified to show sequence similarity to the *Alu* families of SINEs. Refinement of repeat consensus sequence revealed that it is a SINE family whose 5′ region shows strong overlap to 7SL RNA genes (Fig. 1). It is designated *SINE1*-*1_EBu* as SINE1 refers a SINE family with 7SL RNA-derived head. The consensus sequence of *SINE1*-*1_EBu* is 282 bp long. There are 2363 full-length insertions *of SINE1*-*1_EBu* in the hagfish genome, if excluding the 3′ microsatellites composed by AAC trinucleotides.

The 5′ 96-bp sequence of *SINE1*-*1_EBu* shows strong sequence similarity to 7SL RNA genes in humans (Fig. 1b). One copy of 7SL RNA gene was characterized from the hagfish genome (accession number FYBX02009602: 3123756-3,124,041), and it is more similar to *SINE1*-*1_EBu* than to the human 7SL RNA gene. The predicted promoter box A in hagfish is 1 nucleotide different from that in the human genome (GGCGC**A**GTGG and GGCGC**G**GTGG; changes are in bold). The box B is different by 2 nucleotides between human and hagfish, AG**T**TCTG**G**GCT and AG**C**TCTG**C**GCT (changes are in bold), respectively.

**Fig. 2:**
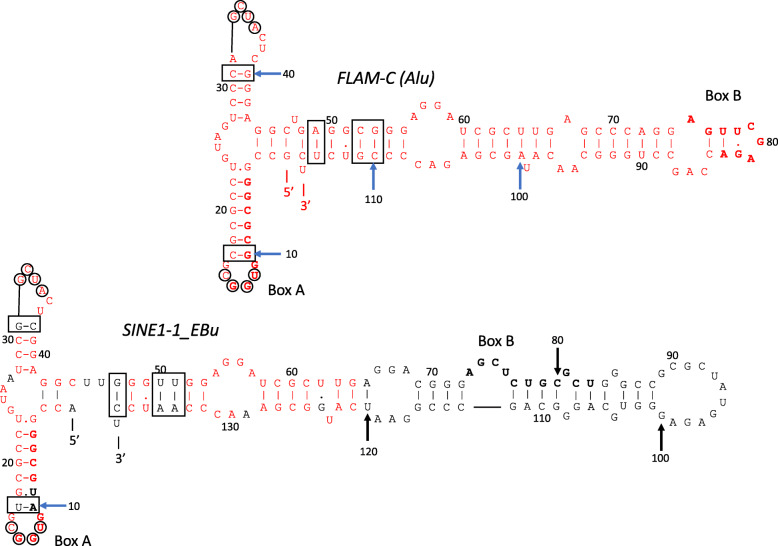
Predicted secondary structures of 7SL RNA-derived regions of *FLAM*-*C* (the ancestor of left monomer of *Alu*) and *SINE1*-*1_EBu*. Nucleotides identical to the corresponding nucleotides in *FLAM*-*C* are colored in red in the structure of *SINE1*-*1_EBu*. Bases in the loops that form tertiary base pairs are circled. Compensatory substitutions are boxed. The nucleotides constituting the promoter box A and box B are in boldface

The sequence 97-140 of *SINE1*-*1_EBu* is similar to the 3′ terminus of 7SL RNA, while it shows less sequence similarity to the human 7SL RNA gene or SINE1 families in Euarchontoglires (Fig. 1b). Despite their sequence differences, *SINE1*-*1_EBu* and *FRAM* show very similar lengths of deletions at the middle of 7SL RNA genes. The 5′ and the 3′ regions of 7SL RNA constitute *Alu* domain. The predicted secondary structure of *SINE1*-*1_EBu* is consistent with the formation of *Alu* domain (Fig. 2). Compensatory substitutions of base-pairing nucleotides are seen and the overall structures are very similar between *FLAM*-*C* and *SINE1*-*1_EBu*. The ability to form *Alu* domain is likely a sequence constraint for SINE1.

### *SINE1-1_EBu* appears mobilized by *RTE*-type non-LTR retrotransposons

The 3′ parts of *SINE1*-*1_EBu* shows some similarity to known *RTE* clade of non-LTR retrotransposons, such as *RTE*-*9_LMi* from the migratory locust *Locusta migratoria* and *RTE*-*2_AFC* from African cichlids (data not shown). Two families (*RTE*-*2_EBu* and *RTE*-*4_EBu*) of non-LTR retrotransposons which show > 90% sequence identity to the parts of *SINE1*-*1_EBu*, were reconstructed from *RTE*-related repeats in the RepeatModeler outputs (Supplementary data S1). There are two and three full-length copies of *RTE*-*2_EBu* and *RTE*-*4_EBu* in the hagfish genome, respectively, in addition to many fragmented copies. However, none of these copies encode intact proteins. Copies of *RTE*-*2_EBu* and *RTE*-*4_EBu* are ~ 93% identical to their respective consensus sequences.

*SINE1*-*1_EBu* was revealed to be a SINE having bipartite *RTE*-derived sequences (Fig. 1c). The sequence 165-231 of *SINE1*-*1_EBu* is almost identical to a part of the 5′ UTR of *RTE*-*2_EBu*, while the sequence 226-274 to the 3′ end of *RTE*-*2_EBu*. Besides, the sequence 137-197 shows a high similarity to a part of the 5′ UTR of *RTE*-*4_EBu*. These sequence similarities strongly support that *SINE1*-*1_EBu* is mobilized by *RTE*-*2_EBu* or its closely related non-LTR retrotransposon family.

### Evolutionary relationships among SINEs and LINEs in the hagfish genome

The average identity of the top 10 copies of *SINE1*-*1_EBu* to the consensus is ~ 99% and thus, *SINE1*-*1_EBu* is a young family. *SINE1*-*1_EBu* generates ~ 17-bp target site duplications (TSDs) upon integration, though the lengths of TSDs are not uniform (Supplementary Fig. S1A). Older copies also show similar lengths of TSDs if allowing a few nucleotide substitutions (data not shown). *SINE1*-*1_EBu* does not show strong target sequence preference. The full-length copies of *RTE*-*2_EBu* are flanked by 16 or 18-bp TSDs (Supplementary Fig. S1B), while the full-length copies of *RTE*-*4_EBu* are by 6 or 19-bp TSDs (Supplementary Fig. S1C).

The sequence 137-274 of *SINE1*-*1_EBu* is similar to the sequence 198-337 of *SINE2*-*1_EBu*. *SINE2*-*1_EBu* has a longer sequence similar to *RTE*-*4_EBu* than *SINE1*-*1_EBu* has (Fig. 1c). This similar sequence corresponds to the sequence 1-90 of *RTE*-*4_EBu*, while *SINE1*-*1_EBu* contains the sequence corresponding to the sequence 35-90 of *RTE*-*4_EBu*. The TSD length of *SINE2*-*1_EBu* is similar to those of *SINE1*-*1_EBu*, *RTE*-*2_EBu* and *RTE*-*4_EBu* (Supplementary Fig. S1D).

**Fig. 3:**
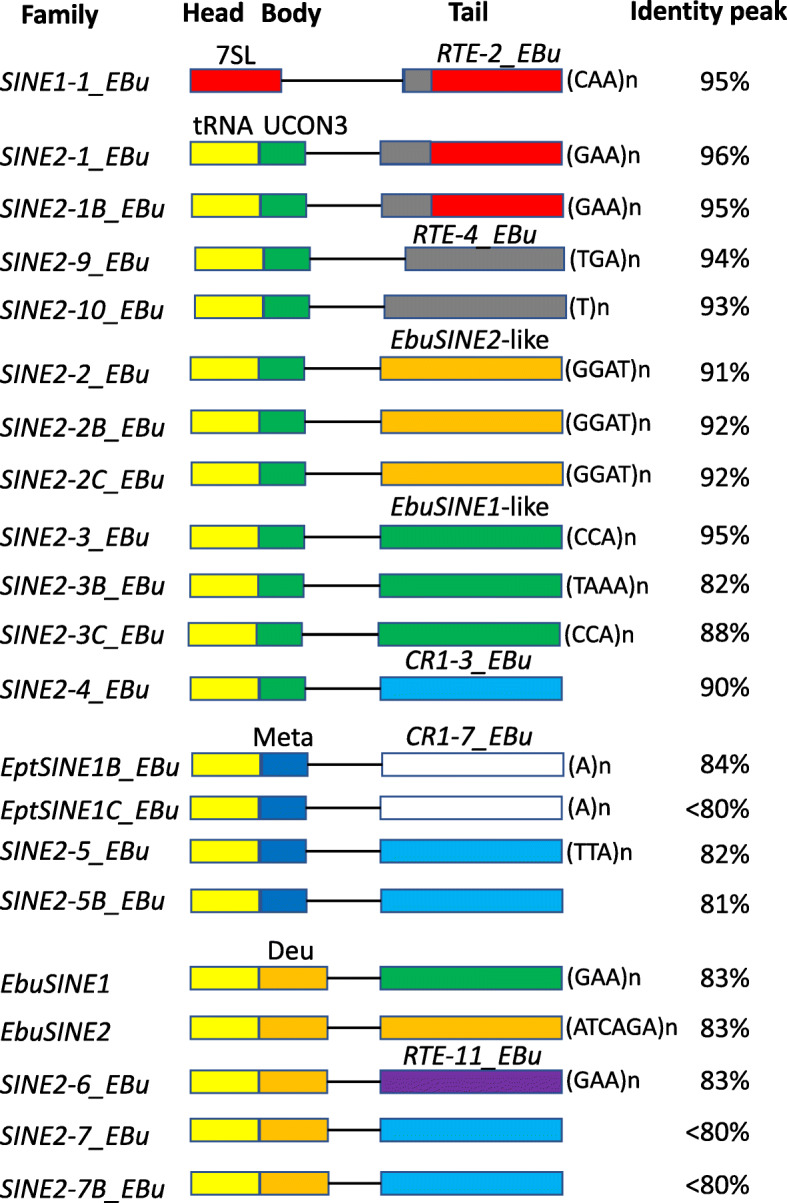
Schematic structures of hagfish SINEs. Two types of head (7SL and tRNA), three groups of body (UCON3, Meta, and Deu), and six groups of tail sequences are observed among the hagfish SINE families. Different groups are shown as boxes in different colors. Boxes are not in scale. The peaks of distribution of sequence identity to the consensus sequences were calculated and shown at the right side

*SINE2*-*1_EBu* has a tRNA-derived sequence at its 5′ terminus (Fig. 3). The sequence following the tRNA-derived sequence shows similarity to *UCON3*. *UCON3* was first found as an ultraconserved element shared among diverse vertebrate genomes [25]. Later, the similarity of *UCON3* to a SINE family from chimaera, designated *UCON3_CM*, clarified that *UCON3* is a part of SINE [26]. The position of sequence similar to *UCON3* in *SINE2*-*1_EBu* is downstream of tRNA-derived sequence and upstream of *RTE*-derived sequence, indicating *UCON3* is a type of conserved body, which is hereafter called UCON3 domain. Censor search against Repbase with UCON3 domain of *SINE2*-*1_EBu* as the query revealed SINE families in various animals, Chordata, Nematoda, Cnidaria, and Xenacoelomorpha, contain the conserved UCON3 domain (Supplementary Fig. S2).

### SINE families from hagfish show the SINE evolution through chimera formation

Besides *SINE2*-*1_EBu*, 17 SINE families whose 5′ termini show similarity to tRNAs were newly characterized (Fig. 3). Some families, such as *SINE2*-*3_EBu*, *SINE2*-*3B_EBu* and *SINE2*-*3C_EBu*, are closely related to each other with > 95% identity among their entire consensus sequences. It should be mentioned that they do not have parent-child relation or whole-part relation. They are independent SINE lineages, which shared their recent ancestor but transposed independently. The sequence alignment suggest that these tRNA-derived regions have several different origins (Supplementary Fig. S3). All SINE2 families found from the hagfish genome contain either of middle “body” regions: Deu, Meta, or UCON3 (Fig. 3 and Supplementary Figs. S2 and S4). *EptSINE1* contains a tRNA-derived head and the middle Meta domain [27]. Comparison with newly characterized *EptSINE1B_EBu* and *EptSINE1C_EBu* revealed that the original *EptSINE1* sequence does not contain its 3′ region. The 3′ region of *EptSINE1B_EBu* and *EptSINE1C_EBu* shows sequence similarity to a newly characterized *CR1*-type non-LTR retrotransposon family, *CR1*-*7_EBu* (Supplementary Fig. S5). *EbuSINE1* and *EbuSINE2* contain a tRNA-derived head and a Deu domain at the middle, but their 3′ regions show no sequence similarity to each other or to any non-LTR retrotransposons [28].

Some newly characterized hagfish SINE families show similarity to *UCON3* (Supplementary Fig. S2). SINE families with UCON3 domain have distinct 3′ tails. *SINE2*-*1_EBu* and *SINE2*-*1B_EBu* contain *RTE*-type tail, almost identical to that of *SINE1*-*1_EBu* (Fig. 1c). *SINE2*-*9_EBu* and *SINE2*-*10_EBu* also contain *RTE*-type tails, but they contain the sequences similar to the 5′ UTR of *RTE*-*4_EBu* and the 3′ UTR of *RTE*-*4_EBu* (Fig. 1c). In other words, these two families of SINEs contain non-autonomous bipartite sequences derived from *RTE*-*4_EBu*.

*SINE2*-*2_EBu*, *SINE2*-*2B_EBu* and *SINE2*-*2C_EBu* have tails similar to that of *EbuSINE2*, despite the fact that *EbuSINE2* contains a Deu domain upstream of the similar tail (Fig. 3 and Supplementary Figs. S4 and S5). *SINE2*-*3_EBu* and *SINE2*-*3B_EBu* have the tail similar to that of *EbuSINE1*, whereas *EbuSINE1* contains a Deu domain (Fig. 3 and Supplementary Figs. S4 and S5). *SINE2*-*4_EBu* has a tail showing similarity to the 3′ UTR of *CR1*-*3_EBu*, and this tail shows similarity to the tails of *SINE2*-*5_EBu* and *SINE2*-*5B_EBu*, which have a Meta domain, and *SINE2*-*7_EBu*, which has a Deu domain (Fig. 3 and Supplementary Figs. S4 and S5). Compared with SINE families with either Meta domain or Deu domain, SINE families with UCON3 domain are younger. It is likely that the recombination between SINE families contributed to the birth of variation of SINE families with UCON3 domain.

### Age and evolution of hagfish SINE families

Including *SINE1*-*1_EBu*, 21 SINE families were characterized from the inshore hagfish genome. The sequence identity of each copy to the consensus sequence is a measure of the age of family of transposable elements. The distributions of sequence identity to the consensus revealed that the youngest SINE family in the hagfish genome is *SINE2*-*1_EBu*, whose peak of identity distribution was between 97 and 96%, followed by *SINE1*-*1_EBu*, *SINE2*-*1B_EBu*, and *SINE2*-*3_EBu*, all of whose peaks were between 96 and 95% (Supplementary Fig. S6). It is also revealed that these 4 SINE families were concurrently active. The concurrent activities of *SINE1*-*1_EBu*, *SINE2*-*1_EBu*, and *SINE2*-*1B_EBu* are consistent with the fact that they appear transposed by the same autonomous non-LTR retrotransposon family, *RTE*-*2_EBu*. At the same time, the sequence similarity in the 3′ tail regions among several SINE families does not guarantee their concurrent activities. *SINE2*-*3B_EBu*, a relative of *SINE2*-*3_EBu* is very old and its peak was between 83 and 82%. *SINE2*-*3B_EBu* and *EbuSINE1* have similar 3′ tails and were concurrently active. It indicates that autonomous non-LTR retrotransposon families were active for the long term with changing counterpart SINE families.

## Discussion

*SINE1*-*1_EBu* is composed by 5 different parts (Fig. 1). The most 5′ region originated from the 5′ region of 7SL RNA, while the second 5′ region originated from the 3′ region of 7SL RNA. These two parts are considered as the head of *SINE1*-*1_EBu*. The central part was derived from the 5′ UTR of *RTE*-*4_EBu*. The two 3′ regions originated from a non-autonomous *RTE*-*2_EBu*, corresponding to the 5′ and 3′ parts of *RTE*-*2_EBu*. The latter three parts are considered as the tail of *SINE1*-*1_EBu*, but the central part can also be considered as the body, considering its independent origin from the 3′ two regions [29].

**Fig. 4:**
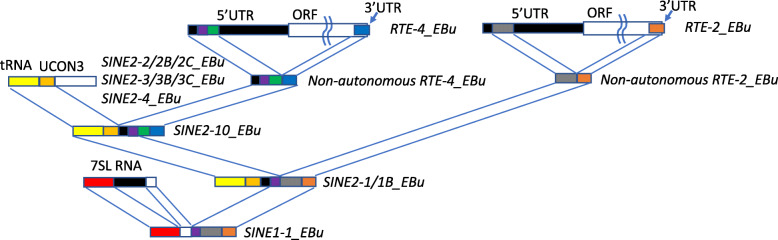
A model of the birth of *SINE1*-*1_EBu*. Internal deletion of *RTE*-*4_EBu* and *RTE*-*2_EBu* generated non-autonomous bipartite retrotransposons. A chimeric SINE family, similar to *SINE2*-*10_EBu*, was generated from a non-autonomous *RTE-4_EBu* derivative and a SINE family having tRNA head and UCON3 domain. The 3′ part of this SINE family was replaced by non-autonomous *RTE-2_EBu* derivative, which may be intact or a part of SINE, generating a SINE family similar to *SINE2–1/1B_EBu*. Finally, the chimera formation between a 7SL RNA and *SINE2–1/1B_EBu*-like SINE family generated *SINE1–1_EBu*. Internal deletion of 7SL RNA occurred either before or after the chimera formation. *SINE1–1_EBu* is composed by 5 parts: 5′ region of 7SL RNA, 3′ region of 7SL RNA, 5′ region of *RTE-4_EBu*, 5′ region of *RTE-2_EBu*, and 3′ region of *RTE-2_EBu*

Based on the findings, it can be hypothesized how *SINE1*-*1_EBu* was born (Fig. 4). An autonomous non-LTR retrotransposon family related to *RTE*-*4_EBu* generated a non-autonomous derivative by the internal deletion. Similar event may have occurred with *RTE*-*2_EBu*. This type of bipartite non-autonomous retrotransposons are common for *RTE*-type non-LTR retrotransposons [29]. The chimera formation of non-autonomous, bipartite *RTE*-*4_EBu* with a SINE2 family, possibly structurally related to *SINE2*-*2/2B/2C_EBu*, *SINE2*-*3/3B/3C_EBu* or *SINE2*-*4_EBu*, generated a SINE2 family which shows the same structure as *SINE2*-*10_EBu*. Such chimera of two SINE families could be generated by DNA recombination. Switching template RNAs during retrotransposition is another possible mechanism of chimeric SINE formation [30]. This family would have been mobilized by *RTE*-*4_EBu*. The chimera formation between this *SINE2*-*10_EBu*-like SINE and a non-autonomous, bipartite *RTE*-*2_EBu* generated a SINE family similar to *SINE2*-*1/1B_EBu*. The chimera formation between this *SINE2*-*1/1B_EBu*-like SINE and 7SL RNA generated a new SINE1 family. Internal deletion of 7SL RNA-derived region either followed or predated this event gave rise to *SINE1*-*1_EBu*. This scenario is simplified the most, and intermediate SINE or non-autonomous families, which have not yet characterized or have been lost completely from the genome, may have contributed to the birth of *SINE1*-*1_EBu*.

*SINE1*-*1_EBu* and *SINE2*-*1/1B_EBu* were concurrently active, which is expected since they appear mobilized by the same autonomous counterpart *RTE*-*2_EBu*. Slightly more recent activity of *SINE2*-*1_EBu* than *SINE1*-*1_EBu* does not contradict to the model, considering that relatively old copies of *SINE2*-*1/1B_EBu* could have contributed to the birth of *SINE1*-*1_EBu*.

*RTE*-type non-LTR retrotransposons often generate non-autonomous derivative families, which lack the internal portion of the autonomous counterpart. These bipartite non-autonomous *RTE* families sometimes generate chimeric retrotransposon families by acquiring 5′ head sequences originated from non-coding RNAs [29]. Most of such SINE families contain the sequence derived from tRNAs, and some contain the sequence from 5S rRNAs. Two reported bird SINE families contain GC-rich heads of unknown origins upstream of bipartite *RTE* sequences [31]. One reported SINE family from budgerigar, called *MeloSINE*, has the 3′ end sequence of 28S rRNA [31]. A SINE family called *PlatSINE1* or *snoRTE* from platypus contains the sequence originated from snoRNAs at its 5′ end [6, 32]. Their uniform chimeric structures among copies support their classification as SINE families. *SINE1*-*1_EBu* is the first reported SINE family containing a 7SL RNA-derived head and bipartite *RTE* sequences.

Theoretically, SINE1 can be born multiple times independently, as SINE2 and SINE3. Multiple independent events of birth of SINE2 are well supported by the different origins of tRNA-derived heads and the very wide distribution of SINE2 [6]. The alignment of head regions of hagfish SINE families also supports several independent origins for their heads (Supplementary Fig. S3). SINE2 families mobilized by various non-LTR retrotransposon families are reported [33]. SINE3 is less abundant. SINE3 was first found from zebrafish [3], and now it is known that SINE3 is present in various vertebrates and some insects (*SINE3*-*1_TC* from the red flour beetle *Tribolium castaneum* and *HaSE3* from a moth *Helicoverpa armigera*, and their related SINE families) [6, 28, 34, 35]. Vertebrate SINE3 families are transposed by the *CR1* clade of non-LTR retrotransposons, *SINE3*-*1_TC* seems mobilized by the *I* clade of non-LTR retrotransposons, and *HaSE3* seems mobilized by the *RTE* clade of non-LTR retrotransposons [29].

*Alu* and *B1* are mobilized by *L1*-type non-LTR retrotransposons [2, 21]. Regarding the nature of SINE mobilization by the transposition machinery of non-LTR retrotransposons, mammalian *L1* is an exception. Mammalian *L1* can mobilize any RNAs with poly A tail [2, 22], including cellular mRNA, RNA of endogenous retroviruses, or even RNA of RNA viruses [36–39]. The 3′ ends of *Alu* and *B1* are not similar to the 3′ ends of *L1* except poly A tails.

All SINEs with 7SL RNA-derived sequences found in euarchontoglires can be considered to be descendants of a single ancestral SINE1 family, born in the common ancestor of Euarchontoglires [7]. The ancestral SINE1 family could have resembled *FLAM*-*A/PB1*, which was an internally deleted derivative of 7SL RNA but had a poly A tail. The internal deletion as well as subsequent deletions/duplications distinguishes SINE families with 7SL RNA-derived sequences from retrocopies of 7SL RNA. *P7SL_Cpo* found from the guinea pig genome corresponds to the full-length 7SL RNA sequence followed by a polyA tail [10]. *P7SL_Cpo* should have originated independently from other SINE1 families, but the possibility that it corresponds to a set of retrocopies of 7SL RNA cannot be excluded. It is known that various types of small RNAs, such as small nuclear RNA (snRNA) or small nucleolar RNA (snoRNA) generate retrocopies which is composed of the full-length or partial RNA sequence and a 3’-poly A tail [30, 38, 40]. A proof that at least one copy of *P7SL_Cpo* is transposition-competent, is needed to establish the classification of *P7SL_Cpo* as a SINE1 family. *P7SL_MD* from marsupials is structurally almost identical to *P7SL_Cpo* despite the sequence differences due to the divergence between these two groups of mammals [8, 9]. There is not yet enough evidence for *P7SL_Cpo* and *P7SL_MD* to be recognized as SINE1 families.

It is obvious that *SINE1*-*1_EBu* was born independently from *Alu* and *B1*. *SINE1*-*1_EBu* appears mobilized by *RTE*, based on the sequence similarity of the 3′ tail of *SINE1*-*1_EBu* with that of *RTE*-*2_EBu*. The internal deletion of 7SL RNA sequence in *SINE1*-*1_EBu* clearly excludes the possibility that it is a chimeric retrocopy of 7SL RNA and *RTE*-related non-LTR retrotransposons. Independent deletion events of the middle region of 7SL RNA, corresponding to the S domain, support no or little functional contribution of the S domain to the SINE proliferation. In contrast, the parallel conservations of base-pairing and the secondary structure of the *Alu* domain indicate the functional importance of the *Alu* domain in the SINE proliferation. The conservation of *Alu* domain is indicated to be linked with the efficient inclusion of *Alu* RNA in the *L1* retrotransposition machinery [24]. Similar mechanism could have selected the conservation of *Alu* domain in the evolution of *SINE1*-*1_EBu*.

## Conclusions

The finding of *SINE1*-*1_EBu*, the first evident SINE1 family outside of Euarchontoglires, reveals the independent, parallel evolution of 7SL RNA-derived SINEs. The conservation of secondary structure of *Alu* domain in independent SINE1 families indicates the functional importance of ternary structure of *Alu* domain bound to SRP9/14 in the proliferation of SINEs.

## Methods

### Identification of SINE and LINE families from the seashore hagfish genome

The genome sequence of inshore hagfish *E. burgeri* (Eburgeri_3.2) were downloaded from NCBI Assembly database (https://www.ncbi.nlm.nih.gov/assembly) on March 14, 2018. RepeatModeler (http://www.repeatmasker.org/RepeatModeler/) and Repbase [6] were used for the initial screening of repetitive families with default parameters. Consensus sequences generated by RepeatModeler with the annotation for either SINE or LINE were chosen to reconstruct refined consensus sequences using the top 10 hits in the Censor search [41] with their 1000-bp flanking sequences at both sides. 7SL RNA gene sequences from the hagfish genome were found using the BLASTN searches using the 7SL RNA gene sequence from *Ciona intestinalis* (accession number: HG323729) as a query.

### Secondary structure prediction

Secondary structure of *FLAM*-*C* was predicted based on that of human 7SL RNA and *AluY* reported in [24]. Secondary structure of *SINE1*-*1_EBu* was predicted based on the sequence alignment and the secondary structure predicted at Web Servers for RNA Secondary Structure Prediction (https://rna.urmc.rochester.edu/RNAstructureWeb/).

## Supplementary information

**Additional file 1: Figure S1.** Target site duplications (TSDs). **Figure S2.** Sequence alignment of UCON3 domains of SINEs. **Figure S3.** Sequence alignment of tRNA-derived head regions of hagfish SINEs. **Figure S4.** Sequence alignments of body domains of hagfish SINEs. **Figure S5.** Sequence alignments of tails of hagfish SINEs. **Figure S6.** Age distribution of hagfish SINE families.

**Additional file 2: Data S1.** Consensus sequences of transposable elements characterized in this study.

## Data Availability

All data generated or analyzed during this study are included in this published article and its supplementary information files. Consensus sequences of transposable elements are also available in Repbase (http://www.girinst.org/repbase/).

## Abbreviations

SINE: Short interspersed element
LTR: Long terminal repeat
snRNA: small nuclear RNA
SRP: Signal recognition particle
TSD: Target site duplication
RTE: Retrotransposon-like element
