# Supplementary material for: Hagfish genome reveals parallel evolution of 7SL RNA-derived SINEs
Source: Mob DNA. 2020 May 22;11:18. doi: 10.1186/s13100-020-00210-2 (PMC7245038; doi:10.1186/s13100-020-00210-2)
Supplement: Supplementary file 1 — Additional file 1: Figure S1. Target site duplications (TSDs). Figure S2. Sequence alignment of UCON3 domains of SINEs. Figure S3. Sequence alignment of tRNA-derived head regions of hagfish SINEs. Figure S4. Sequence alignments of body domains of hagfish SINEs. Figure S5. Sequence alignments of tails of hagfish SINEs. Figure S6. Age distribution of hagfish SINE families. [file 13100_2020_210_MOESM1_ESM.pdf]

(A) *SINE1-1_EBu*

```
TGTTCATAGTGTTTTGTAATAACATACCGGGCGTAGTGGCGTGCGCCTGTAATCCGGCTACTCGGAGGCT//GCGACCCCTAACGGGAGCAGCCGAAAGACGAACAACAAC------------AGTGTTTTGTAATAACATTAACATT
ATACATCATGTTTATATAGCTTGATACCGGGCGTAGTGGCGTGCGCCTGTAATCCGGCTACTCGGAGGCT//GCGACCCCTAACGGGAGCAGCCGAAAGACGAACAACAAC------------ATGTTTATATAGCTTGGATAATTCA
TGTGCTGTACATAGTTTTTGGCCCCACCGGGCGTAGTGGCGTGCGCCTGTAATCCGGCTACTCGGAGGCT//GCGACCCCTAACGGGAGCAGCCGAAAGACGAACAACAACAACAAC------ACATAGTTTTTGGCCCCATCTCTCA
ACCCCTCACTTCACTGATGGGAAAAACCGGGCGTAGTGGCGTGCGCCTGTAATCCGGCTACTCGGAGGCT//GCGACCCCTAACGGGAGCAGCCGAAAGACGAACAACAACAACA--------ACTTCACTGATGGGAAAATTAAGAT
NNNNNATATATATATGTGGGGTTTAGCCGGGCGTAGTGACGTGCGCCTGTAATCCGGCTACTCGGAGGCT//GCGACCCCTAACGGGAGCAGCCGAAAGACGAACAACAAC------------ATGTGTGGGGTCTGCTTACACATTG
TTGTCAGGTTGACATGGCAGATGAGACCGGGCGTAGTGGCGTGCGCCTGTAATCCGGCTACTCGGAGGCT//GCGACCCCTAACGGGAGCAGCCGAAAGACGAACAACA--------------ACATGGCAGATGAGAGGAGAAGGCT
ATCTAAAGTTAACCACTAGTCATGCACCGGGCGTAGTGGCGTGCGCCTGTAATCCGGCTACTCGGAGGCT//GCGACCCCTAACGGGAGCAGCCGAAAGACGAACAACAAC------------AACCATTAGTCATGCAGTCATGTTC
CCCTATACATTAATTGGGGTCATGAACCGGGCGTAGTGGCGTGCGCCTGTAATTCGGCTACTCGGAGGCT//GCGACCCCTAACGGGAGCAGCCGAAAGACGAACAACAACAAC---------ATTAATTGGGGTCATGCTGATCCTC
CTAATGACACGAATAGGAGCTTGAAACCGGGCGTAGTGGCGTGCGCCTGTAATCCGGCGACTCGGAGGGT//GCGACCCCTAACGGGAGCAGCCGAAAGACGAACAACAACA-----------ACGAATAGGAGCTTGAACTCAGGAA
GAAGGTTGACATCATGTACTTTCTAACCGGGCGTAGTGGCGTGCGCCTGTAATCCGGCTACTCGGAGGCT//GCGACCCCTAACGGGAGCAGCCGAAAGACGAACAACAACAACA--------ACATCATGTACTTTCTAAATTGTAG
TCCTCCATAGGAAATGCTCTGCTGGACCGGGCGTAGTGGCGTGCGCCTGTAATCCGGCTACTCGGAGGCT//GTGGCGACCCCTAACGGGAGCAGCCGAAAGACGAACAACACA---------AGGAAATGCTCTGCTGGGTGTGGTG
AATTTATGATGTAGGAGTTGTGTAAACCGGGCGTAGTGGCGTGCGCCTGTAATCCGGCTACTCGGAGGCT//GCGACCCCTAAAGGGAGCAGCCGAAAGACGAACAACAAC------------ATGTAGGTGTTGTGTAAACAAATAG
CACCGATATATATTGTAGCCTTCTGACCGGGCGTAGTGGCGTGCGCCTGTAATCCAGCTACTCGGAGGCT//GTGGCGACCCCTAACGGGAGCAGCCGAAAGACGAACAAC------------ATATCTTGTAGCCTTCTAATCCTGT
TGTGTTGTCACCTTTTTCGTATTCTACCGGGCGTAGTGGCGTGCGCCTGTAATCCGGCTACTCGGAGGCT//GCGACCCCTCACGGGAGCAGCCGAAAGACGAACAACAACA-----------ACCTTTTTAGTATTCCAAGGTAGGG
GGGGAAATAATACAATATAAGTGGCACCAGGCGTAGTGGCGTGCGCCTGTAATCCGGCTACTCGGAGGCT//GCGACCCCTAACGGGAGCAGCCGAAAGACGAACAACAACAACAACA-----ATACAATATAAGTGGCGTAGGTTCA
AACTTTGACTTTGGTTTCACTTTTGACCGGGCGTAGTGGTGTGCGCCTGTAATCCGGCTACTCGGAGGCT//GCGACCCCTAACGGGAGCAGCCAAAAGACGAACAACAACAACACA------ACTTTGGTTTCACTTTTTAGTTTTC
AACAGCACATGATGTCGATAACTAAACCGGGCGTAGTGGCGTGCGCCTGTAATCCGGCTACTCGGAGGCT//GCGACCCCTAACGGGAGCAGCCGAAAGACGAACAACAACAACAACAACA--ACATGATGTCGATAACTAATTTAAC
AAAATGCATACAACGCCGGATTGAAACCAGGCGTAGTGGCGTGCGCCTGCAATCCGGCTACTCGGAGGCT//GCGACCCCTAACGGGAGCAGCCGAAAGACGAACAACAACAACAAC------ATACAACGCCGGATTGAACAGTTTA
TCACATTATACATGGGTCAGTGTTCACCGGGCGTAGTGGCGTGCGCCTGTAATCCGGCTACTCGGAGGCT//GCGACCCCTAACGGGAGCAGCCGAAAGATGAACAACCA-------------ACATGGGTCAGTGTTCAGCTCTCCA
ACTTCTCAACTTGTGACATGAATATACCGGGCGTAGTGGCGTGCGCCTGTAATCCGGCTACTCGGAGGCT//GCGACCCCTAACGGGAGCAGCCGAAAGACAAACAACAACAACAACAA----GTGACATGAATGGAAAAACCTCACG
```

(B) *RTE-2_EBu*

```
GGATCCGTTAGGAGTGCTTGAGTTAAACCCAGCCACTGTGGTTGCACAAGTTAGTGCACTCTCAGCGCCG//GCAACCCCTAATGGAAGCAGCCGAATGAAGAAGAAGAAG------------AGGAGTGCTTGAGTTAGCTCAAGAG
TATTAAATGAGATGTGATGACAAAAAACCCAGCCACTGTGGTTGCACAAGTTAGTGCACTCTCAGTGCCG//GCGACCCCTAACGGGAGCAGCCAAAATAAGA--------------------AGATGTGATGACAAAATAAATTGAG
GTGAATCATTCAATGTTCTTCGTCCAACCCAGCCACTGTGGTTGCACAAGTTAGTGCACTCAGTGCGGGT//GCGACCCCTAATGGGAGCAGCCAAAAGAAGAAGA-----------------ATTCAATGTTCTTCCTCCGTAAAAA
```

(C) *RTE-4_EBu*

```
ACCCGTTGCTTCACTGAATATGGTTCCGGCGTGTGCCCCAGTTTAGAATAGGTACACCGTACCCCTGTGT//CCCACTCTTGCAGGGAAAATGGCCTTTAAAA-TGCCCTTTGTGTGTT----GTTGCTTCGTTGAATATGGGAAGGG
GGAAATTTTCTTCTCATTCATCCACCTGGTGTGTGCCACAGTTTAGAATAGGTACACAGTATCCCTGCGT//CCCCCCTCGAGCAGGGGAAATGGCCTTAAAAATTCCTGTTGTGTGTGTTG-TCATCCCTGTTTCCTACCTGTGTAC
```

(D) *SINE2-1_EBu*

```
TCCTTACCCTGGCCTAATATATAATGCCTGGTGTTGGTGCAGTGGTTAGGGCAGCGGCTTGGGATCCAAG//GTGACCCCTAACGGGAGCAGCCGAAAGAAGAAGAAGAAGA-----------CCCTGGCCTAATATATCCCAAATTT
CCCCACATCCCAGTCGTAGTTAGGAGCCTGGTGTTGGCGTAGTGGTTAGGGCAGCGGCTTGGGATCCAAG//GCGACCCCTAACGGGAGCAGCCGAAAGAAGAAGAAGAAGAAGAT-------CCAGTCGTAGTTAGGGTTAAAAGGA
TACCATGCTCCTGACAGAGTGATGAGCCTGGTGTTGGCGCAGTGGTTAGGGCAGCGGCTTGGGATCCAAG//GCGACCCCTAACGGGAGCAGCCGAAAGAAGAAGAAGAA-------------GCTCCTGACAGAGTGATGGTGCTGC
ATTAATACGCTAAGCCATATTTTATGCCTGGTGTTGGCGCAGTGGTTAGGGCAGCGGCTTGGGATCCAAG//GCGACCCCTAACGGGAGCAGCCGAAAGAAGAAGAAGAAGAAG---------ACGCTAAGCCATATTTGGTGGACGT
ATTGGTGGATGATGTATTTGGCACAGCCTGGTGTTGGCGCAGTGGTTAGGGCAGCGGCTTGGGATCCAAA//GCGACCCCTAACGGGAGCAGCCGAAAGAAGAAGAAG---------------ATTGGTGGATGATGTAGAGATATAT
AATTAGGGAGGTTGCAGCCGCTCAAGCCTGGTGTTGGCGCAGTGGTTAGGGCAGCGGCTTGGGATCCAAA//GCGACCCCTAACGGGAGCAGCCGAAAGAAGAAGAAGAA-------------GAGGTTGCAGCCGCTCTTAAGAGAT
GAAAACACTATTCATAACTTCAGAAGCCTGGTGTTGGCGCAGTGGTTAGGGCAGCGGCTTGGGATCCAAA//GCGACCCTTAACGGGAGCAGCCGAAAGAAGAAGAAGAAGAG----------ATTCATAACTTCAGAAAATCACGAA
CCATTTATAATAGATACTTCTTCAAGCCTGGTGTTGGCGCAGTGGTTAGGGCAGCGGCTTGGGATCCAAG//GCGACCCCTAACGGGAGCAGCCGAAAGAAGAAGAAGAAGAAGAAGAAGAAGACTTCTTCATGGCGAGGCGATTGCG
GTGCTACTTAATTCAAGATTGTGAAGCCTGGTGTTGGCGCAGTGGTTAGGGCAGCGGCTTGGGATCCAAA//GCGACCCCTAACGGGAGCAGCCGAAAGAAGAAGAAGAAG------------ACTTAATTCAAGATTGTGGCAACTG
ACCATACTCTGTGCAGAGCATTGAAGCCTGGTGTTGGCGCAGTGGTTAGGGCAGCGGCTTGGGATCCAAG//GCGACCCCTAACGGGAGCAGCCGAAAGAGGAAGAAGAAG------------ACTCTGTGCAGAGCATTGGTGCTGC
TTGTATATGCAATCAACTTTGTAAAGCCTGGTGTTGGCGCAGTGGTTAGGGCAGTGGCTTGGGATCCAAA//GCGACCCCTAACGGGAGCAGCCGAAAGAAGAGGAAG---------------ATGCAATCAACAATGGGAACACATG
ATGTGCATATGTATACCATCATGTAGCCTGGTGTTGGCGCAGTGGTTAGGGCAGCGGCTTGGGATCCAAA//GCGACCCCTAACGGGAGCAGCCGAAAGAAGAAGAAGAAG------------ATGTATACCATCATGTATTATCATT
AATATCATGCTATCTATCATTCTTAGCCTGGTGTTGGCGCAGTGGTTAGGGCAGCGGCTTGGGATCCAAA//GCGACCCCTAACGGGAGCAGCCGAAAGAAGAAGAAGAA-------------GCTATATATCATTCTTGGGCCAATC
AGGTCTGCTTTTTGAACTTCAAAAAGCCTGGTGTTGGCGCAGTGGTTAGGGCAGCGGCTTGGGATCCAAA//GCGACCCCTAACGGGAGCAGCTGAAAGAAGAAGAAGAATA-----------TGCTTTTTGAACTTCAAGTGTCAGT
CACATTATACAGTGTATTACACGAAGCCTGGTGTTGGCGCAGTGGTTAGGGCAGCGGCTTGGGATCCAAA//GCGACCCCTAACGGGAGCAGCCAAAAGAAGAAGAAGAAG------------ATTATACAGTGTATTACACACACAT
TAGTTGGGACTGTTATGCCCAAGGAGCCTGGTGTTGGCGCAGTGGTTAGGGCAGTGGCTTGGGATCCAAG//GCGACCCCTAACGGGAGCAGCCGAAAGAAGAAGAAGAAGAAGAAGAAG---GACTGTTATGCCCAACTTATGTTTT
CAGGACAGATACCGGAGGATTGGATGCCTGGTGTTGGCGCAGTGGTTAGGGCAGCGGCTTGGGATCCAAA//GCGACCCCTAACGGGAGCAGCCGAAAGAAGAAGA-----------------AGATACCGGAGGATTGGAGAGTAGC
AGATTCGTCAGTCAACTTTGTATAAGCCTGGTGTTGGCGCCGTGGTTAGGGCAGCGGCTTGGGATCCAAG//GCGACCCCTAACGGGAGCAGCCGAAAGAGGAAGAAG---------------ATTCGTCAGTCAACTTTGTATAAGC
ATACATACATATATTGTGGACCGGAGCCTGGTGTTGGCGCAGTGGTTAGGGCAGCGGCTTGGGATCCAAG//GCGACCCCTAACGGGAGCAGCCGAAAGAAGAAGAAGAAG------------ATGTATTGTGGGCCGGGGGTCGGTG
AACAGCTACATGGGCATTCACCGAAGCCTGGTGTTGGCGCAGTGGTTAGGGCAGCGGCTTGGGATCCAAA//GCGACCCCAAACGGGAGCAGCCGAAAGAAGAAGAAGAA-------------GGGCATTCACCAAAATGGCACGCCA
```

Figure S1. Target site duplications (TSDs). TSDs are shown in red. (A) *SINE1-1_EBu* and (D) *SINE2-1_EBu*, only the representative 20 copies are shown. (B) *RTE-2_EBu* and (C) *RTE-4_EBu*, all full-length copies are shown.

```
UCON3_[78-131]          CCAG-CAGGGTTAACTCAGCCCTTCATCCTTCTAA-GGTAGATAAATTGAGTTCCA Euteleostomi
UCON3_CM_[64-116]       CCGG--TGGGTCCACTCAGCCCTTCACCCTTTTGA-GGTCGATAAAATGAGTACCA Callorhynchus (shark)
SINE2-1_EBu_[88-140]    CCAG--GGGGTTGACTCAGCCTGTCATCCTTCCGA-GGTCGGCGAAATGAGTACCA Eptatretus (hagfish)
SINE2-1B_EBu_[91-143]   CCAG--GAGGTTGACTCAGCCTGTCATCCTTCCGA-GGTCGGCGAAGTGAGTACCA
SINE2-2_EBu_[88-142]    CCAGGTGGGGTTGACTCGGCCTGTCATCCTTCCGA-GGTCGGCAAAATGAGTGCCA
SINE2-2B_EBu_[90-144]   CCAGGTGGGGTTAACTCAGCCTGTCATCCTTCCGA-GGTCGGCAAAATGAGTGCCA
SINE2-2C_EBu_[88-142]   CCAGGTGGGGTTGACTCTGCCTGTCATCCTTCCGA-GGTCGGCAAAATGAGTGCCA
SINE2-3_EBu_[87-139]    CCAG--GGGGTTGACTCAGCCTGTCATCCTTCCGA-GGTCGGCGAAATGAGTACCA
SINE2-3B_EBu_[90-144]   CCAGGTAGGGTTGACTCAGCCTGTCATCCTTCCGA-GGTCGGCAAAATGAGTACCA
SINE2-3C_EBu_[87-141]   CCAGGTGGGGTTGACTCAGCCTGTCATCCTTCTGA-GGTTGGCGAAATGAGTACCA
SINE2-4_EBu_[88-140]    CCAG--GGGGTTGACTCAGCCTGTCATCCTTCCGA-GGTCGGCGAAATGAGTACCA
SINE2-9_EBu_[85-139]    TCAG-GGGGGTTGACTCAGCCTGTCATCCTTCCGAAGGTCGGTGAAATGAGCACCA
SINE2-10_EBu_[86-138]   CCAG--GGGGTTGACTCAGCCTGTCATCCTTCCGA-GGTCGGTGAAATGAGTACCA
SINE2-5_PM_[64-113]     -----GGGGGTTGACTCAGCCCATCATCCCTCTGG-GGTCGATAAAATGAGTACCA Petromyzon (lamprey)
SINE2-1_CrH_[84-131]    -------AGGTTGACTCAGCCTTCCATCCTTCCGA-GGTCGGTAAAATGAGGACCC Crotalus (snake)
SINE2-1_NAm_[67-113]    -------GTGCTCACCAAGCCTTTCATCCCTCCGG-GGTCGATAAATTG-GTACCA Necator (hookworm)
SINE2-2_NAm_[72-118]    -------GTGCAAACCAAGCCTTTCATCCCTCCGG-GGTCGATAAATTG-GTACCA
SINE2-15_ADi_[104-151]  -------AAGTTCACTCAGCTTTCCATCCTTCCGA-GGTCGGTAAAATGAGTACCA Acropora (coral)
SINE2-21_ADi_[127-174]  -------AAGTTCACTCAGCTTTCCATCCATTCGT-GGTGGGTAAAATGAGTACCA
SINE2-1_XB_[98-144]     -------AGGCCAACTCAGCCTTCCATCCTTCCGG-GGTCGGTGAA-TGAGTACCG Xenoturbella
SINE2-2_NW_[68-114]     -------GGGTCCACTCAGCCTTTCATCC-ACTGG-GGTCGATAAAATGAGTACCA Nemertoderma
```

Figure S2. Sequence alignment of UCON3 domains of SINEs. Nucleotides identical to those of *UCON3* are colored in red. The positions inside of Repbase entries are shown in parentheses. Abbreviations for organism names are as follows: ADi, *Acropora digitifera* (coral); CM, *Callorhinchus milii* (elephant shark); CrH, *Crotalus horridus*, timber rattlesnake; NAm, *Necator americanus* (hookworm); NW, *Nemertoderma westbladi* (acora); PM, *Petromyzon marinus* (sea lamprey), XB, *Xenoturbella bocki*.

```
                                                 A-Box                                            B-Box
SINE2-1_EBu_[1-70]     ----GCCTGGT--GTTGG-------CGCAGTGGTTAGGG---CAGCGGCTTGGGATCCAAG-AGGTCCTGAGTTSAA-----GCCCCGTTGG
SINE2-1B_EBu_[1-70]    ----GCCTGGT--GTTGG-------CGTAGTGGTTAGGG---TAGCGGCTTGGGATCCAAA-AGGTCCTGAGTTCAA-----GCCCCGTTGG
SINE2-9_EBu_[1-70]     ---AGCCTGGT--G-TGG-------CTCAGTGGTTAAGG---CGGTGGCTTGGGATCCTTG-GGGTCCTGAGTTCGA-----GCCCCGCTGG
SINE2-10_EBu_[1-71]    ---AGCCTGGT--GTTGG-------CTTAGTGGTTAGGG---TGGCTGGCTTGGGATCCTA-GGGTCCTGAGTTTGA-----GCCCCGTTTT
SINE2-2_EBu_[1-72]     --ATGCCTGGT--GTTGG-------CTTAATGGTTAGGG---TGGTGGGCTTGGGATCCTA-GGATCCTGAGTTCAA-----ATCCCGCTCG
SINE2-2B_EBu_[1-74]    ATATGCCTGGT--GTTGG-------CTTAATGGTTAGGG---TGGCGGGCTTGGGATCCTA-GGGTCCTGAGTTCAA-----ATCCCGCTCA
SINE2-2C_EBu_[1-72]    --ATGCCTGGT--GTTGG-------CTTAATGGTTAGGG---TGGTGGGCTTAGGATCCGA-GGATCCTGAGTTCAA-----ATCCCACTCG
SINE2-3_EBu_[1-70]     ---AGCCTGGT--GTTGG-------CTCAGTGGTTAGGG---TGGC-GGCTTGGGATCCTA-GGGTCCTGAGTTCAA-----GCCCCGTCGG
SINE2-3B_EBu_[1-73]    -CAGGCCTGGT--GTTGG-------CTTAACGGTTAAGG---TGGCTGGCTTAAGATCCTA-GGGTCTTGAGTTCAA-----GCCCTTGTCG
SINE2-3C_EBu_[1-72]    --AAGCCTGGT--GTTGG-------CTTAGTGGTTAGGG---TGGCTGGCTTGGGATCTTA-GGGTCCTGAGTTTGA-----GCCCTGTTGG
SINE2-4_EBu_[1-73]     TTGAACCTGGT--GTTGG-------CTTAGCGGTTAGGG---TGGT-GGCTTGGGATCCTA-GGGTCCTGAGTTCAA-----GCCCCGTTGG
EptSINE1_[1-72]        -----CCAGACTTGGTGG-------TGTAATGGTTGGAG---CGTCCGCTCCGAGTCTGGGAAGGTCGGCGGTTCAA-----ACCCCGGCCG
EptSINE1B_EBu_[1-71]   -----CCAGACTCGGTGG-------TGTAATGGTTAGAG---CGTCCGCTCCGAGTCTGGG-AGGTCGGCGGTTCAA-----ACCCCGGCCG
EptSINE1C_EBu_[1-74]   -------AGACTCGGTGG-------CATAATGGTTAGAG---CGTCCGCTCTGAGTCTGGG-AGGTCGGCGGTTCGATTCCGACCCTGGCCG
SINE2-5_EBu_[1-72]     ----ACCAGACTCGGTGG-------CGTAATGGTTAGAG---CGTCCGCTCTGAGTCTGGG-AGGTCGGCGGTTCGA-----ACCCCGGCCG
SINE2-5B_EBu_[1-70]    ------CAGACTCGGTGG-------TGTAATGGTTGGAG---CGTCCGCTCTGAGTCTGGG-AGGTCGGTGGTTCAA-----ACCCCGGCCG
EbuSINE1_[1-72]        ---GGGCTGACAGGATGG-------CCTAGAGGTACGCA---CACTCACCTCTAAGCTGTC-TAGCC-TGGGTTTGA-----ATCCCGACCC
EbuSINE2_[1-74]        --------GGCAGGATGGCCTAAACCCTAGAGGCACGCA---CACTCRCCTCTAAGCTGTC-TAGCC-TGGGTTTGA-----ATCCTGACCC
SINE2-6_EBu_[1-71]     ----GGCTGGCAGGATGG-------CCAAGAGGTACGCA---CACTCGCCTCTAAGCTGTC-TAGCC-TGGGTTTGA-----ATCCCGACCC
SINE2-7_EBu_[1-60]     ----------------AG-------CTCAGTTGGTATGC---GTGCTGCCTCTGAGCTGTC-TGGTCATGGATTTGT-----TCCCCTGTGG
SINE2-7B_EBu_[1-59]    ----------------AG-------CTCAGTTGGTAGCG---TGCTCGCCATTGAGCTGTC-TAGTC-TGGGTTTGA-----TCCCCGACGG
SINE2-11_EBu_[1-75]    ---GAGCTGGCTGTTTGG-------TCGAGTGGTTAAAGACTTGTTTCTTTCTCCACTGGC-TGGTG-TGGGTTTGA-----TTCCCATGGC
SINE2-8_EBu_[1-67]     ------ATGGT--GGTGG--------CTTGTGGTGAGGA---CACTGGACTGGCATCCCAG-AGGTTGTGGGTTCAA-----ATCCAGCTGA
```

Figure S3. Sequence alignment of tRNA-derived head regions of hagfish SINEs. Two conserved regions for transcription promoter (A- and B-boxes) are indicated. The positions inside of Repbase entries are shown in parentheses.

Deu domain

```
EbuSINE1_[126-267]     GTGCCGTCCCTCGGAT--GGACGTTAAACTGGGCGTCCCGTCTGCCGGCATTAGTTGGTG
EbuSINE2_[128-268]     GTGCCGTCCCTCAGAT--GGACGTTAAACT-GGCGTCCCGTCTGCCGGCATTAGTTGGTG
SINE2-6_EBu_[125-266]  GTGCCGTCCCTCGGAT--GGACGTTAAACTGGGCGTCCCGTCTGCCGGCATTAGTTGGTG
SINE2-7_EBu_[72-211]   ATGCCGTCCTTCAGATGGGGACGTAAAGCCGGCTGTCCCGTCTGC---ATTTAGGTTGTG
AmnSINE1_HS_[213-343]  GTGCCGTCTTTCGGAT-GAGACGTAAAACC-GAGGTCCTGACCAC----------TTGCG

EbuSINE1_[126-267]     GACGTTAAAGATCCCACGGTGTCCTTCGCGAAGAGTAGGCGAGCTATCGCCGGCACCCTG
EbuSINE2_[128-268]     GACGTTAAAGATCCCACGGTGTCCTTCGCGAAGAGWAGGCGAGCTATCGCCGGCACCTTG
SINE2-6_EBu_[125-266]  GACGTTAAAGATCCCACGGTGTCCTTCGCGAAGAGTAGGCGAGCTATCGCCGGCACCATG
SINE2-7_EBu_[72-211]   GACGTTAAAGATCCCACGGTATCTCTGAA-AAGAGTAGGCCATTGTGTGCCGGTACCCTG
AmnSINE1_HS_[213-343]  GTCATTAAAGATCCCATGGCACTTTTCGT-AAGAGTAGGGGTGTTAACCCCGGTGTCCTG

EbuSINE1_[126-267]     AACAAATTCCAAATTCCTGCCCTA
EbuSINE2_[128-268]     AACAAATTACAAATTCYTGCCCTG
SINE2-6_EBu_[125-266]  AACAAATTCCAAATTCCTGCCCTA
SINE2-7_EBu_[72-211]   GCTAAATTCCCTGCCTAGAGATTA
AmnSINE1_HS_[213-343]  GCCAAATTCCAATTCGGGTAATTA
```

Meta domain

```
EptSINE1_[83-150]      AAGACTTTAAAAATGGTACGTGCTGCTTCCTTGCTTGATGCTCAGCATTTAAAAGGATAAGAGCACGG
EptSINE1B_EBu_[82-149] AAGACTTTAAAAATGGTACGTGCTGCTTCCTTGCTTGACGTTCAGCATTTAAAAGGATAAGAGCATGG
EptSINE1C_EBu_[85-151] AAGACTTTAAAAATGGTACATGCTGCTTCCTTGCTTGACGCTCAGCATTTGAAAGGAT-AGAGCATGG
Meta-domain consensus  AAGACTTTAAAAATGGTACTTGCTGCTTCCCTGCTTGGCGCTCAGCATT--AAGGGGTTAGAGTAAGG
```

Figure S4. Sequence alignments of body domains of hagfish SINEs. Nucleotides identical to those of one representative sequence are colored in red. The positions inside of Repbase entries are shown in parentheses. Abbreviations for organism names are as follows: HS, *Homo sapiens* (human). Sequences without the abbreviations shown above are families from the seashore hagfish.

RTE-11_EBu-like

```
RTE-38_CPB_[263-304]      GAACATCAGGCCTGGCCAGCCGGTTTGTAAAAGTTGTGCCAA------------------
SINE2-6_EBu_[276-385]     GAACGTCAGGCCTGGCCAGCCGGTTCGTGAAAGACTTGGGCCAAGCTTCCCCTGGCCAAC
RTE-11_EBu_[1017-1073]    -----------------------------------------------------GGCCGAC

RTE-38_CPB_[263-304]      --------------------------------------------------
SINE2-6_EBu_[276-385]     CCCACAAGAACGTGGGAATAATTGTGGCCGTTAAGAAGAAGAAGAAGAAG
RTE-11_EBu_[1017-1073]    CCCACAAGAAGGTGGGATTAATTGGGGCCATGAAGTAGTAGTAGTAGTAG
```

CR1-3_EBu-like

```
SINE2-4_EBu_[212-268]     GGCGCAGGCATGATGGGCCGAATGGCCTCCTCCTGCGCATAA-TTTCCTTGTAA-TCCTT
SINE2-5_EBu_[178-273]     GTGCAGGCTTTGATGGGCCTAATGGCCTCCTCCTGCGTCGAAG-TTCCTTGTAA-GGGAA
SINE2-5B_EBu_[172-234]    GCACAGGCTTTGATGGGCCTAATGGCCTCCTCCTGCGTTGAAG-TTCCTTGTAA-GGGAA
SINE2-7_EBu_[373-421]     AGCACAGGCATGATGGGCCAAATGGCCTCCTCCTGTGTCTAAGTTTCCT-----------
SINE2-7B_EBu_[313-365]    GGCACAGGCATGATGGGCCAAATGGCCTCCTCCTGTGTCTAAGTTTCCTTGCA-------
CR1-3_EBu_[3949-4008]     CGCGCAGGCATGATGGGCTGAATGGCCTCCACCTGTGTTGGA-TTTCCTTGTTATTCCTT
CR1-4_CM_[4458-4528]      GGCACAGACTCGATGGGCCGAATGGCCTCCTTCTGTGCCGTA-ACATTCTATGA-ATCTA
CR1-1_CM_[4063-4123]      GGGGCAGGCWCGAGGGGCCGAATGGCCTACTCCTGCTCCTAA-TTCTTATGTTC-TTATG
CR1-11_CM_[211-266]       GGAACAGGCTCGATGGGCTGAATGGCCTCCTCCTGTTCCTGT-TTTCCT----A-TGTTC
CR1-5_CM_[4387-4455]      GGCACAGACACGATGGGCCGAATGGCCTCCTTCTGTGCTGTA-TAATTCTATGA-TTCTA
CR1-1_LCh_[3984-4037]     ---GCAGGCTTGTTGGGCCTAATGGCCTTTTCCTGTTCCTAA-AATTTCTTATG-TTCT-
```

CR1-7_EBu-like

```
EptSINE1C_EBu [365-410]  TGTGTTGCAGCTCCTCCATGAGCCTTAGACCCT-GTAGAAAAAAAAA
EptSINE1B_EBu_[367-412]  TGTGTGGCAGCTCCACTACGAGCCTTAGATCCT-GTAAAAAAAAAAA
CR1-7_EBu [2400-2445]    TGTCATTTAGCTCCTCCATGAGCCT-AGACCCTTGTATAAAAAAAAA
```

EbuSINE1-like

```
EbuSINE1_[277-347]        GGGCATTGCATCAGCGGCACAGCCGCGCCCTCAGCCAATRAT-GCTACCCCGCAGCGTTGTGCTG-CATACGA
SINE2-3_EBu_[164-234]     GGGCACTGCATCAGCGACACAGTCGCGCTTCAAGAAATGATA-GCTACCCCGGCAGCTAAGCTGC-CATACGA
SINE2-3B_EBu_[158-228]    GGGCACTGCATCAGCGGCACAGCTGCACCCCCAGAAAATGAT-GCAGCCAAGCAGCCACAGGCTG-CATACGA
SINE2-3C_EBu_[156-226]    GGGCACAGCATCAGCGGCACAGCCGCGCCCCCAAGAAATGATAGCTACCCGGCAGCCAA--GCTGCCATATGC
```

EbuSINE2-like

```
SINE2-2_EBu_[197-253]     CCAAGGAGACTGCTTAGGCAGCACCAACGCTCTG-CACAGAGTATGGTCCCAATGGAT
SINE2-2B_EBu_[199-255]    CCAAGGAGACTGCTTAGGCAGCACCAACGCTCTG-CACAGAGTATGGTCCCAATGGAT
SINE2-2C_EBu_[197-253]    CCAAGGAGACTGCTTCGGCAGCACCAACGCTCTG-CACAGAGTATGGTCCCAGTGGAT
EbuSINE2_[314-370]        CCAAG-AAACTGCTTCGGCAGCACTGACGCTCTGTCACAGAGTATGGTACATCAGAAT
```

Figure S5. Sequence alignments of tails of hagfish SINEs. Nucleotides identical to those of one representative sequence are colored in red. The positions inside of Repbase entries are shown in parentheses. Abbreviations for organism names are as follows: CM, *Callorhinchus milii* (elephant shark); CPB, *Chrysemys picta bellii* (western painted turtle); LCh, *Latimeria chalumnae* (coelacanth). Sequences without the abbreviations shown above are families from the seashore hagfish.

A

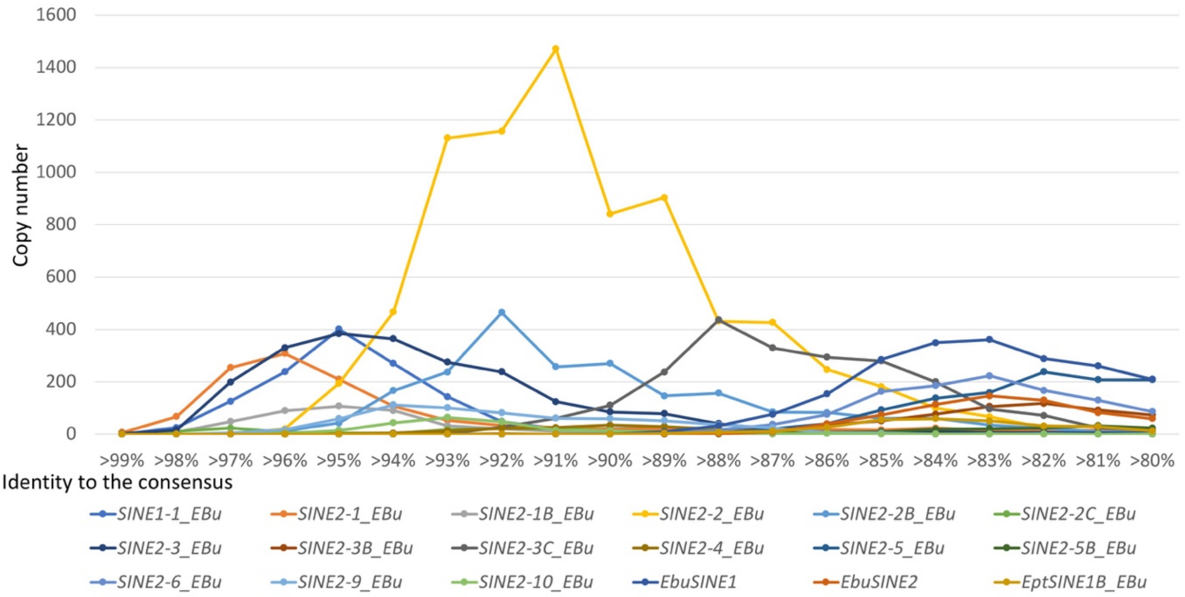

B

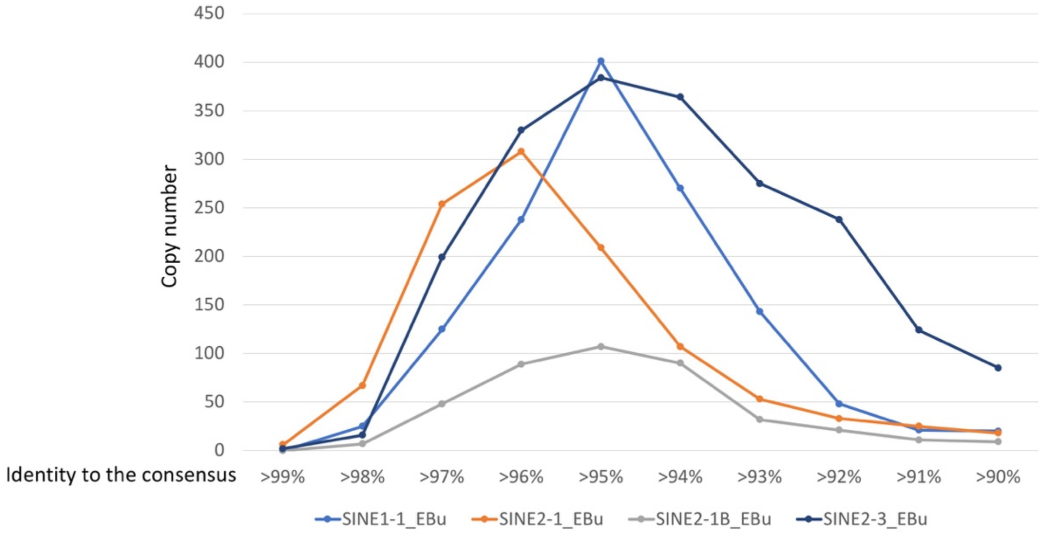

Figure S6. Age distribution of hagfish SINE families. (A) All families except four families whose copy numbers are lower than 100 (*SINE2-7_EBu*, *SINE2-7B_EBu*, *SINE2-8_EBu*, and *EptSINE1*). (B) The most recently active 4 families.
