# Supplementary material for: Hagfish genome reveals parallel evolution of 7SL RNA-derived SINEs
Source: Mob DNA. 2020 May 22;11:18. doi: 10.1186/s13100-020-00210-2 (PMC7245038; doi:10.1186/s13100-020-00210-2)
Supplement: Supplementary file 2 — Additional file 2: Data S1. Consensus sequences of transposable elements characterized in this study. [file 13100_2020_210_MOESM2_ESM.pdf]

Data S1. Consensus sequences of transposable elements characterized in this study.

>CR1-3_EBu
ggcgggcagtggtgacggtgcgacgggkgcgggcagtggcggaggtgcggcgggkgcaggcagtggcgga
ggtgcagcgggkgctggcagtggcggmggtgcagcatgggtgcaggcagtggtggaggtgcagtgggtgc
gggcagtggcggaggtgcagcgggtgcaggcagtggtggaggtgcagcgggtgcaggcagtggtggaggt
gcagtgggtgcaggcagtggtggaggtgcagcgggtgcaggcagtggtggaggtgcagcaatataaattg
gtagaaatgaggtatgggagacagtttttgtgggtgatgagacaattaaatatgtagattcttcattctg
caggaaggatcgtagacatagaattctgtgcagttttgaaggagcgggagttagggacattgaaggaagg
gtaaacgatattgtatcgggtcatggcaggaagaagttggttgtggtacacctcggtgcgaatgatgtag
ggaaagtttactcagaggttttgaaaaatagatacaggatattgggtaagcggctgaaagcaaagggttg
tgaggtcatgttttctgggattctcccaagattaggaaacaatatagaaattatgagcagagcaatagac
gtcaaccaatggctggaagaatggtgtagggaggaaggttttacatttaagaagcagtgggaatcattta
ggggacaaagggagtgtttccggaatgatggccgtatgttaaatagaaagggggcagcaaggtttgcagt
gggaattgaggaaggaattcaggctttttttaggtcagcggagggtggggaagctattaaggaaggaacg
gatgatgtaattgtgatgagtaaggccaggcaggaaaatagaatttcaggaagggaaaagttaaatatat
tctataccaatgccagaagtttaaataataagatggaagagcttgaagccaaagtagatagcgaggagta
cgatatagttgcagtgagtgagacatggtttaaggaggagagtaactggaggacaggattagaaggctac
aaggtgtatcggtgtgataggaaggagagaataggaggaggagtagcaatctgggtaaaggacagcattg
catcaagagaaaggggtgacattaaggaaggaataaatgttgaggattctgtatgggtagagattagaga
ttgtaaaaacagtaagatactggtaggatgcttttatagggctccgggtgtgaaggaagaggaggaagga
ggagtacatgaagaattcaaaagggcatgtggtaagggaagagtattgattatggggattttaacttgc
cagggatagattgggtccaggaacagggaaagggcagagcagatgaggatttcctagttttagtccagga
ttgctttttaacccagtttgtagataagcccacgagggggagtgcagtgttagacctccttttaagtaac
gatcccaatatggtggaggaggtggaggttggggagcatttaggagcaagtgaccataacattgtgtgtg
caaagattctgttgagggttagagttcaggatagtagggtaaggctattagatttcaggaaggcaaactt
tgagggtatgaggagagaattgggtgatgtagattgggagactctaacaacaggacaatcagcatcagag
aaatgggaaaccttcaaggaccaaatgtgtagagtgcagagtaagtacattccaatgaggtgtaaaacta
gaagcagaaagagaccagggtggttgagcagtgagataaggaatgctattaaggagaagcagaaggcatt
tattaggtttaaaataacaggactggagttagatttatatcactatcgaagtaaacagaggcaagttaag
aaaataactcggcaagccaaaagagagtatgagaaggacatggctaagaatattaaacataatagcaagg
ctttctttaagtatatcaggggaaaagaacaggttaggaccagtgtgggcccattgaggaatagcaccgg
gagagtggttagtgatgaaagtgagatggcaggcttattaaacaggtatttttcttcaacttttacacaa
gaacagtcaggagaattgccaatagctgagagtatctatcgaggaggtgaggaagggcttttacagaaga
tccaggttggagtggaggaagtaaaggaacagctaggaaatctaagagwagataaggctccgggtccaga
taatatgcaaccgagagtattgatggaggtggctgagcaggtaagcgagatgttaacggatattttcaat
agctcactggagtcaggacaggtaccggaggattggagagtagcaaatgtaacaccattatttaagaaag
gatccagagaagaattgggaaactatagacctgttagtttgacttcagttgtggggaaggtgttggagac
attaattaaggaccagatgaggaaccatttaaacaaatacaaactaattaaaggtagtcagcatgggttt
accaaaggtagttcctgtttaacaaatcttttagagttctatgaagcagtgtctgattgggtggatgagg
gaaaggcagtggacatagtatatctagactttaagaaggcatttgataaggtaccacatcggaggttgtt
agccaaagtgagggcttgtggagtggctggtcaggtagctaattggattgcaaactggcttagtgatagg
aagcaaagagtggctgtgagtgggaggatgtcttgctgggaggatgtaagtagcggggtacctcagggat
cagtactcggaccattactattcantatttacatcaacgatttggatagtggggtgaagagtaagctttc
caaatttgcagatgacacaaagcttggaggtaaggtggatagcagaggggggtggtgatcagatacaggaa
agtattgatacttgtatggattgggcaaaagattggcagatggaatttaacctgagcaagtgtaaggtgt
tggggatgggaaagaacaatgaaaatagagattacaggatgcaaggggcaattttggagcgtgtcacaca
ggagaaggatctgggggtggtaatagatacgggaggtaagcaggcagcacagtgccaggcagctacaggt
aaagcaaatagggttttaggttgtatttggaggggggatcatttataaatcaaaagaggtggtgttaacat
tgtacaggaatttagtgaggccacatccagaatattgtgtgcagttttggtcaccacaattcaggaagga
catagatgccatagagagggttcagcgcagggctactagattgattccgggcctagctagactcagctat
gaggagcggctgaaggagacagggctttatacactagaaaggaggcggttacgaggagatatgatagaga
tgtttaagataatgaaaggtatagataaaataagtgcagatgaattattcaacagagtagacagtgatag
aaccaggggccatagcctgagagtaaagaagaggagagttaagacggtagtaaggcaggggacttttact
cagagagtagttaatgcatggaatggcctccctggaaaggtagtggcagcagaaaaagtagataagttta
aattagaattagataggtacctggaagttatggagatagaggggtatggggatgttgacaggacttttag
gtagtttgagcattgatgggtgattgtgtgcatgtgtacggagtgctgttggtgggggtcttgtcaaggg
gggttgtgtgtttttaaccaagcccatgccccctcccctgaggggaagaaaagaagaaaaaaaaacaaaa
acaaaaaaacaaaaacaaaaacatttttatttcattaattaaaaactttttttgcatttcttccttgg
atcttgtgtagatgggttggtgtggtgttcgcgcaggcatgatgggctgaatggcctccacctgtgttgg
atttccttgttattccttg
>CR1-7_EBu
catagccgagcagctaagtcattctgtgaaatgactggacttactcagtctgtttcttttccaacacaca
tctccccaaatggcaaccattcatactggaccttatcatgtcaaacttttcctcaaacttgtcctgctcc
tcctcagctcctcgaggctcttctgaccacgtgttggtaaaaggtggtcgtctctttgacagttattaga
gaacccccaaggtgcagacgtgtggaattggctaagctgattggtctggcctaaaatgtgctctctccag
tcaagactggtccatcatcacatcagctccaacattgactgctcctggaatttcttctacaaagactaat
ttctcttcttcacaagttcatcccatcccgcattcaacttttctgtccatcatctcgaccctggtacact
gatgcgtgtggtgaggccatcgcaagaaaacaacttgcattctcctcctggaaggcaaaccctactgatg
gcaacctgcagtctctccgcacagctcggaaccattgtgtatctgtcctacgaagagccagggcccaaca
tcaggctcacctgtgatccaaattctccagcctctcaccatcctccaaagcctggtgggagctggtcagg
tctgtgtctggtacacattccccttccattccttccctcctaaaggacactgtcttcactcaaaggcca
aagtcccaaatccgtcccttcctgtacccaccctttccccattacagaatgttccctacagcatgtgga
cttctcccctgccatagtggagaagtttcttacccagctcgacacctcttctgcaactggtcctgatggc
ataagtgctctaatcctcaggaactgtgcttcagtcctatcatacccctcctcttctttcacaggtccttc
ttggatgggcaacttcctccatcttggaaagttgcaaacatcactcctatccataagaaaggttcaaaac

tgatcccctcaactacagacctatcagccttctccccatcatcagcaaagtaatggagtccatcttgacg
gtctcttctctaactcgctaatctctgacaaccagtttggcttccgacctggccattccaccttggatat
gttactccttctgacacagcagtggatggatgccctgaaccacaagcaagaggttagggccatctctctg
gatatctctcgagcctttgacactgtctggcatccagccttgatttctaagttgtcttcatttggtattc
agggccccctgctcaagtggttgtctgatttttttcctctatgagcagaaacagcaggtggcccttgatgg
tgtcctttcccatgatctttcccttcaggctggggttccacaaggcagcatcttaggacctacattattc
ttgatctatatcaatgatctgtctgattctcttgaaaaccaactttaccattttgccgatgactccaccc
tgtgcagaacgatacatcacacttgtgaaaggctggccgctgctgaatccctttttgtcagacctggacaa
gataagccgctggtcagctacttggaacatgatgttcaatgcggcaagtcacacgctctcacactgtctc
tccacaaggatcggcaatcaaactatccaatccacttctctggacagccactggaagaggtgcagtccct
ggaactccttggcctcactatctgccataacctttcatgggaggatcatatcaccaagctagcatcaaag
gccagtcgccgtctgggtattctccgtagagctaaggcattcctgagccgcaccgagctcatcacggtgt
acaaggctttcatatggagctccatggagtatggctccccactctgggctggtgctcctgcttcccacct
tgctcctcttgaggcagtcaaaaacaaggccttccgcaccattggatctcaagacaggaggcggattccc
aaggactgtcactctcccaccggtggcaagttggtgggctctctattttatacaagcttacatctggcct
tgctcctcctgccctctcctacctctgccctccccaagccaccccaggtcacacgcgttcctcaagaaac
cctcacctgctcaccctcccaaagtccaggctggttgcccacctgcattcattcgtccccttattctccc
aattgtggaatcgtctccctgtgtccgttgtctcatcctcctcacttcagtctttcaaatgcgctgccca
tcactacctcatgcccccatgtcttctacacacgctgattctttcccattctgttcctctctgggccatt
gttactcgtttttgtgttattcctcctttcctccttgtgtatgtccacccccccatacccttttggtagt
ttgtgaacctgctgccctttgtcatttagctcctccatgagcctagacccttgtataaaaaaaaaaa
>RTE-11_EBu
ttttaagcagagataggataaattttggaagggaacagtaagttgtattgtgggtttgtggatctggaga
aagcatatgataggttccwagggaggtggtgtattggtgtctgagaagaatgagggctgaaagactgtaa
gggtagttgaggtgatgtatgaaggggctaagacatcagtcagaacaggaatggttgctcagaggcattt
gagataaaggtaggtttacatcaagggtcagctcttagtccttttgtgtttgtggtggtgatggatgtgt
tgagtgagggagcaagaagagggttaccttgggaaatgttgtttgcagatgatttggtggtggtgtcaga
ctctgaggagggacttcaggatgcaatggttgcatggcaggaaagtttggaaagtggggataagtgtggg
gaaacagaggtaatggtgagcaagagagagggaagagaggatgtggatattgggatagggcaggcaggga
gttaaagcaggtgggaaaattcaagtacctgggttcttcgaggggggtggggtgtgggggttgaggcagg
gtggagtaagtggaggagtgtttctggggtggtcatgatcaaaagattcggttaggctgagggcaaagat
ttacaaaatggtaataagaccagttctcctgtatggagcagaggcctgggccttgagaaaggaagagaga
gattgctgcagaggatggagatgcggatgctgagagggctgatggggatctctctgagggaaaggaagag
agtgggcaggagtaagggacattagcgaaaaggcacgagaggcacgtctgcgttggttggggcatgtgct
caggagggaggagggaatgtggtcaggcaggtctttgatatggatgcagttggcaggaggagaaggggca
gaccaaaattgaggtggaaggaggtggtggagagggacatgagagaggtgggggtgcagagagaggatgc
tcgggatagaggggtgtggagagggaaaactcatgcggccgaccccacaagaaggtgggattaattgggg
ccatgaagtagtagtagtagtagtgtagtagtagtagtagtagtagtagtagta
>RTE-2_EBu
aacccagccactgtggttgcacaagttagtgcactctcagcgccggtcccaagcccggataaatggggag
ggttgcgtcaggaagggcatccggcgtaaaacctgtgccaaatcaatatgcggatcataaaacggatttc
cataccggatcggtcgaggcccgggttaccaacgaccgccaccggtactgttggccagcagggtaccggt
ggaaactgtgctactgctgggcgaaggaggagaaggagagggggaaggcgtgtccagaggcagtgggaga
ggaggaagggtagaagtgtggaggtgagagttggaactttgaatgttggtactatgactggcaaagggcg
agagttggctgatatgatggagaggagaaaggtggacatactgtgtgtacaagagaccaagtggatgggg
tgtaaggctaggagcatcggaggtggtttcaaactgttctaccatggtgtggatgggaggagaaatggtg
tnggggtaatcctgaaggaggattatgttaagagggtattggaggtgaggagagtgtctgaccgagtgat
gagtgtgaagttggaaattgaaggggtgatgatgaatgtcatcagtgcatatgccccgcaagtggggtgt
gagatggaggagaaagaagatttctggagtgagttagatgaagtggtggagagtgtatccaaggaagaaa
gagtggtgattggagcggatttcaatgggcatgttggggaagggaatagagatgatgaggaggtgatggg
taggtacggtgtcaacgagaggaatgtggaagggcagatggtggtggattttgcaaaaagaatggaaatg
gcggtggtaaatacgtattttaagaagaaggagcacagggtgacgtacaagagtggaggaaggtgcacac
aggtggactacgtcttaagcaggagatgcaacctgaaagagattagagactgtaaggtggtggcagggga
gagtgtagctagacagcatcggatggtggtttgtaggatgattttggtggtgaggttgaggaagagagtg
agggctgaaccaaggaccagatggtggaagctgaaggaggaagactgttgcgtgaaattcagggaggagg
tgagacaggtgttggatggtggtaaagaggtgtgggacgactgggcaactactgcagaagtggtgaggga
gatagctaggaaggtacttggtgtgacatctggacagaggaaggaagacaaggagacttggtggtggaac
gaggaagtacaggaaagtataaggaagaagaggttggcgaaaaagaattgggatagccagagagatgaag
aaagtagacaggagtacaaggagtggcggcgcaaggcgaagagagaggtggcgaaggctaaggaaaaggc
atatggcgagctgtatgagaagttggatacaaaggaagggaaaaggatttgtaccgattggccagacag
agggaccgagctgggaaggatgtgctgcaggttagggtgataaaggacgcagatgggaatgtgctgacaa
gcgaggagagtgtgttgagaaggtggatggagtactttgaggagctgatgaatgaagaaaatgagagaga
gagaaggttggaagaggtggagatagtgaatcaggaagtgcggtggattagtaaggatgaagtgaggact
gctatgaagaggatgaagagtggaaaggcggttggcccagatgatataccagtggaggcatggaagtgtt
taggagagatggcagtggggtttttaaccagattgtttaataaaatcttggaaagtgagaggatgccкga
ggagtggagattaagtgtactggtaccgattttaagaataagggtgatgtgcagagctgtggtaattac
agagggataaagttgttaagccacaccatgaagttgtgggaaagagtagtggaagctaggttgagaggag
aggtgatgatttgtgagcagcaatatggttttatgccaggaaagagcaccacagatgcgatgtttgcttt
gagaatgttgatggagaagtatagggaaggccagaaggagttgcattgtgtgtttgtggatttagagaaa
gcttatgacagggtgccaagagaggagttgtggtattgtatgaggaagtcgggagtggtagagaagtatg
tgagggtggtgcaggatatgtatgaggacagtgtgacagcggtgaggtgtgcggtaggaatgacggaccg
gtttaaggtagaggtgggattgcaccaaggatcggctctgagccctttcctgtttgcaatggtgatggac
aggttgacggacgagatcagacaggagtctccatggactatgatgttcgcggatgacatcgtgatctgta
gtgagagtagggagcaggctgaggcgagcctcgagaggtggaggtacgcactggagagaaggggaatgat
agtcagtnggagtaagacggagtacatgtgcgtaaatgagagggagggtggtggaatggtgcggttgcaa
ggagttgaggtggtgaaggtggatgggtttaaatacttgggatcaactgtccaaagtaacggagagtgtg
gtagagaagtgaagaagagagtgcaggcaggatggagtgggtggagaagagtttcaggagtgatttgtga

tagaagggtatctgcaagagtgaaagggaaagtatacaagacggtagtgagaccagctatgttgtatggt
ttggaggcggtggcgctgacaaaaagacaggaagcggagctggaggtggcagagttaaagatgctgcgat
tctcgttgggagtgacgaggatggacaggattagaaatgagtatattagagggacagcgcaggtaggacg
gtttggagacaaagtgagagaggcgagattgagatggtttggacatgtgcacaggagggatgcggggtat
atcgggagaaggatgctgaggatggagccgccaggtaggaggagaagaggaaggccaaagaggaggttta
tggatgcggtgagggcggacatgcgggtggttggtgtgacaatggaagatgtggaggacagagcgaaatg
gagacggatgatccgctgtggcgacccctaacgggagcagccaaaagaagaaga
>RTE-4_EBu
ccggtgtgtgccccggtttagaataggtacaccgtatccctgcgtgtcgtaagaggcgactaaaagggtt
ggcgtcaggaagggcatccggccataaaacattgcctcaaaaagggaaccatgatgagtacaattatata
tgggcagggtacagcccgaagtaccccgtgggggtcctcaccccctctcgtcaaaaatcaaggtggggaa
cccgatcgaagcaagggaggaaattactccattcatgacgaccaaatgtctgatgccccacgtggagatc
ggtttctgaggtttgggacatggaatgtagggacaatgacagggagatcaggagaagtggtggaagtatt
ggtcagaagaagagtggacatatgttgtgtgcaggagacgagatggaagggaagtggtgcgagaatggtg
aagggtaggcagggacagaagtacaagtttgtgtggcaagggtgcccggaaggtgtgtatggtgttggtg
tcctgttttctgaggagtttgtagacagtgtggtgagtgtgactagagtgagtgaaaggttgatgatggt
gagaatggtgattggaaagctcttggtgaatgtcatctcgggatatgccccgcaggtcggtcggagcgat
gaagagaaggacaagttctggtgtgtgatagagaagttgatggaaaatgtgaaagatgaggaagttgttg
tggtaggaggagacttgaatggacatgtgggaaggagtacagatggttttgagggagtccatgggggtta
cggatatggtgtgaggaatggagaaggtgaaaggattttggagtttgctgatggagctggtttgttgatt
tgtaatactcaattccagaaagcggataataagcttgttacatacacgtcaggtgggtcgacgacaattg
ttgactacctgatggtgcgtaggcgagatagggggaacctccgggacacaaaggtgatcccgggggagga
ggctgtttcacagcaccacttggttgtctgcgacataaggatgaaaggtgcgaggagagctcggcgggtg
aagtaccaaccgaggatgaaggtgtggaggttgaaagaggtgacagtgaaggaggcattaagagagaggt
tgagtatggtagaggatgatgtggttgagcttgaaagtggttggatgtcaatgaaagatagtctgttggg
agtcgttggtgaggtatgtggatggacaaaaggaccaccgaggcactcggaaacatggtggtgggatgat
gaagttggaaagaggattgacgagaagaggaagaagttcaaggagtggcagaaggcgaagggtacggtgg
cagaagaggcagcgtatgaaagttataaggcagccaagaaggctgcaaagaaagaggtagcaaaggcgaa
ggaggcacataggaaaatgctcggagaacgtttggattcggaagagggtcagagagcggtatttaggatt
gcaaagcagatagcgaaagagaggtgcgatgtgacgggggtgagttgcttgaaggatgaagtgggacaaa
tcgttgtggacccagatggaattaaaggaagatggaagagatatatggaaaggctgttgaatgtagagaa
tgaatgggatggcaacatagagagcaacccaattttaggaccggcagagaagataactgagaaggaagtt
gaggaagcgataagagcaatgaagagtagaaaagcaggagggccgacgggagttgtgggagatatgttga
aggcagcgggaagttggggtgtgaagagaatgacagagatctgtaatctggtggtgaaagaaggccgcat
tccagtggattgggaactgagtaccctcgtccctctgtacaaggggaaaggtgaccctctggactgtggc
tcgtatagagcgataaagttactggagcatggaatgaaggtattggaaagggtgttggagaagaggataa
ggaagaaggtgaagattgacgaaatgcagtttggatttatgccaggaagaggaacgacagatgcgatttt
tattgtaaggcagttgcaggagaagtacatggagaagaggaagaagttgtttcttgggtttgttgatctg
gagaaagcattcgatagggttccacgcgaagtagtgacatgggcattgaggaagttaggagtggaggaat
ggttgatcagagtagtgatggcaatgtacgagagagcgcgcacagctgtaaggacgaaggatgggaacag
tggtgagtttgaggtgaaggtgggagtgcatcaggggtcggtactaagtccactactatttgtagttgtg
atggaggtgctggctcggagcgcaaaggagggtttaccgtgggagctgttgtatgcagatgatttggtgt
tgattgcggagagtagggatggactgaaggagaagatgaataagtggaaggaatgtatggaggcaaaagg
tttgaaggtgaatatcggaaagacgaaggtgatggtaagtggtaaaacatgtggtgaggtggaaaggact
gggaagtggccgtgtgctgtgtgcaggaagggtgtcggagtgaactccattcagtgtacaatgtgcgctg
agtgggttcatcgaaaatgtagtggtgtgagagggtcattaaccagtgtggcagctacattcaagtgtaa
ggtgtgcatagagggagttgcagatggaggaaatgttgagttggatcttggagatggcgtgaagttggaa
ggtgtgaaaacattttgctacctaggagacatgctgaatggagaaggtggatcagactcggcgacggttg
ctggagtgagatgtgcatggaagaagtttcgggaactgagtggcgtgctgacgaggaggggagtgtcgtt
gaagttgaagggaaaggtgtatgctacatgtgtgagaagtgctatgatttatgggagcgagacatgggta
atgaacgtagagcagcaacgaaggctggaaagggtggaaatgcgaatggttcggtggatgtgcggaatat
cgttgagagagagaggaagacaaatgatgaattgcggaagatgatgggaatagagcctgtgatggatgtt
gtgaagagaaacaggttgagatggttgggtcatgtgttgaggaaggatgaaagtgactgggtgagaagag
tgatggaaatgaatgtagaaggaagtagaggacggggaagaccgagaaagacttggctaaaggtggtgga
agaagaaatgtgggtaaggggggttaacaaggggagatgcagaaaatagggcaaagtggagaagattgtca
tggggcccgcaaggctaacccccgctctagcagggaaaatggccttaaaaatgcctgttgttgttg
>SINE1-1_EBu
accgggcgtagtggcgtgcgcctgtaatccggctactcggaggcttgggttggaggatcgcttgaggacg
ggagctctgcgctgggccgcgctatgagagggtgtagggcagcccggaatcatggcgaaacccaatcctt
atccctgtgtcgtaagaggcgaccagggttgcgtcaggaagggcatccggcgtaaaacctgtgccaaatc
agtatgcggatcataacggatgatccgctgtggcgacccctaacgggagcagccgaaagacgaacaacaa
ca
>SINE2-1_EBu
gcctggtgttggcgcagtggttagggcagcggcttgggatccaagaggtcctgagttsaagccccgttgg
gccactgaattaatacaccaggggggttgactcagcctgtcatccttccgaggtcggcgaaatgagtacca
gtgtactgggccggtgtataccccggttaaaataggtgtaccatatccctgtgtcgtaagaggcgaccag
ggttgcgtcaggaagggcatccggcgtaaaacatgtgccaaatcackatgcggatcatacggatgatccg
ctgtggcgacccctaacgggagcagccgaaagaagaagaaga
>SINE2-1B_EBu
agcctggtgttggcgtagtggttagggtagcggcttgggatccaaaaggtcctgagttcaagccccgttg
gccgcttgaattaatacacaccaggaggttgactcagcctgtcatccttccgaggtcggcgaagtgagta
ccagtgtactgggcccggtgtatgccccggttagaataggtgtaccatatccctgtgtcgtaagaggcga
ccagggttgcgtcaggaagggcatccggcgtaaaacctgtgccaaatcattatgcggatcataacggatg
atccgctgtggcgacccctaacgggagcagccgaaagaagaagaagaaga
>SINE2-2_EBu
atgcctggtgttggcttaatggttagggtggtgggcttgggatcctaggatcctgagttcaaatcccgct
cggctgttgaattaataccaggtggggttgactcggcctgtcatccttccgaggtcggcaaaatgagtgc

cagcttgctggtatcctgtgtcggagtggcgacccgtccaggattgtgcccaatagccaaggagactgct
taggcagcaccaacgctctgcacagagtatggtcccaatggatggatggatg
>SINE2-2B_EBu
atatgcctggtgttggcttaatggttagggtggcgggcttgggatcctagggtcctgagttcaaatcccg
ctcagctgttgaattaacaccaggtggggttaactcagcctgtcatccttccgaggtcggcaaaatgagt
gccagcttgctggtatactgtgtcggagtggcgacccgtccaggattgtgcccaatagccaaggagactg
cttaggcagcaccaacgctctgcacagagtatggtcccaatggatggatggat
>SINE2-2C_EBu
atgcctggtgttggcttaatggttagggtggtgggcttaggatccgaggatcctgagttcaaatcccact
cggctgttgaattaataccaggtggggttgactctgcctgtcatccttccgaggtcggcaaaatgagtgc
cagcwtgctggtatcctgtgtcggagtggcgacccgtccaggattgtgcccaatagccaaggagactgct
tcggcagcaccaacgctctgcacagagtatggtcccagtggatggatggatg
>SINE2-3_EBu
agcctggtgttggctcagtggttagggtggcggcttgggatcctagggtcctgagttcaagccccgtcgg
ccgctgaattaatacaccaggggggttgactcagcctgtcatccttccgaggtcggcgaaatgagtaccag
ttccagtgtgtgtactggtagaggggcactgcatcagcgacacagtcgcgcttcaagaaatgatagctac
cccggcagctaagctgccatacgaccaccac
>SINE2-3B_EBu
caggcctggtgttggcttaacggttaaggtggctggcttaagatcctagggtcttgagttcaagcccttg
tcgtsctgttgaattaacaccaggtagggttgactcagcctgtcatccttccgaggtcggcaaaatgagt
accagcatgctggtaacgggcactgcatcagcggcacagctgcacccccagaaaatgatgcagccaagca
gccacaggctgcatacgaataaataaataa
>SINE2-3C_EBu
aagcctggtgttggcttagtggttagggtggctggcttgggatcttagggtcctgagtttgagccctgtt
ggctgctgaattaacaccaggtggggttgactcagcctgtcatccttctgaggttggcgaaatgagtacc
agtgtactggtagaggggcacagcatcagcggcacagccgcgcccccaagaaatgatagctacccggcag
ccaagctgccatatgcaagaagaagaa
>SINE2-4_EBu
ttgaacctggtgttggcttagcggttagggtggtggcttgggatcctagggtcctgagttcaagccccgt
tggctgctgaattaacaccaggggggttgactcagcctgtcatccttccgaggtcggcgaaatgagtacca
gtgtagcagccaaaaggctgcacacgaagggaaggggtattgacggatttattaaatctggtattggtgt
ggcgcaggcatgatgggccgaatggcctcctcctgcgcataatttccttgtaatcctt
>SINE2-5_EBu
accagactcggtggcgtaatggttagagcgtccgctctgagtctgggaggtcggcggttcgaaccccggc
cgagtcataccaaagactttaaaaatggtacgtgctgcttccttgcttgacgctcagcatttaaaaagga
tagagcacggacaaacaaccataggttggcaataatagtgcaggctttgatgggcctaatggcctcctcc
tgcgtcgaagttccttgtaagggaactcatattattattattattatcatcattattattatt
>SINE2-5B_EBu
cagactcggtggtgtaatggttggagcgtccgctctgagtctgggaggtcggtggttcaaaccccggccg
agtcataccaaagactttaaaaatggtacgtgctgcttccttgcttgatgctcagcaatttaaaaggata
gagcatggacaagcacaagttggcaaccattgcacaggctttgatgggcctaatggcctcctcctgcgtt
gaagttccttgtaagggaactcat
>SINE2-6_EBu
ggctggcaggatggccaagaggtacgcacactcgcctctaagctgtctagcctgggtttgaatcccgacc
cagctataaactgtcatcctggtgtttcacaggcagggtgattcatcaatgtgtgtgccgtccctcggat
ggacgttaaactgggcgtcccgtctgccggcattagttggtggacgttaaagatcccacggtgtccttcg
cgaagagtaggcgagctatcgccggcaccatgaacaaattccaaattcctgccctaaccttagtcgaacg
tcaggcctggccagccggttcgtgaaagacttgggccaagcttcccctggccaaccccacaagaacgtgg
gaataattgtggccgttaagaagaagaagaagaaga
>SINE2-7_EBu
tcccctgtggagtcgaatccgactaaaactgccgtcctggtgcagtgccggcgggtgctgcatctcggag
catgccgtccttcagatggggacgtaaagccggctgtcccgtctgcatttaggttgtggacgttaaagat
cccacggtatctctgaaaagagtaggccattgtgtgccggtaccctggctaaattccctgcctagagatt
aaatgggattgagcagttataaagggatgtggatgttaagttagattaagttaatggtaagaatggggtt
agtacatagaagataagtaaattgatagattaagttaatggggtgtggatacagcacaggcatgatgggc
caaatggcctcctcctgtgt
>SINE2-7B_EBu
agctcagttggtagcgtgctcgccattgagctgtctagtctgggtttgatccccgacggggtcggactta
acaaaactgccgttctggtgcagtgccagcaggggcgctgcatctcgagcacgccgtccttcggatggg
gatgtaaagccggctgtcccatctgcatttaggttgtggacgttaaagatcccacggtatctttcaaaaa
gaggccattgtgtgccggtaccctggccaaattccctgcttggggttagctgtataattagcactacctg
cagagtaggtgggagtgctggggtgtggatatggcacaggcatgatgggccaaatggcctcctcctgtgt
ctaagtttccttgca
>SINE2-9_EBu
agcctggtgtggctcagtggttaaggcggtggcttgggatccttggggtcctgagttcgagccccgctgg
ctgctgagttagcatcaggggggttgactcagcctgtcatccttccgaaggtcggtgaaatgagcaccag
tatactggtaattatccctgcagggtcgaaggcgactaaagggttggcgtcaggaagggcatccggccat
aaaaacattgccacaagaaagggtaccatgattggagcccgcaaaaagctgacccctgctcaggataaa
ggctttcaaatggtgtgatgatga
>SINE2-10_EBu
agcctggtgttggcttagtggttagggtggctggcttgggatcctagggtcctgagtttgagccccgttt
tccactgaattaacaccaggggggttgactcagcctgtcatccttccgaggtcggtgaaatgagtaccagt
gtactggtcccggtgtatgccccggttagaataggtgtaccatatccctgtgtgtcgtaagaggtgacca
gggttggcgtcaggaagggcatccggccataaaaacattgcctcaagaaggaaccatgattgcatggag
cccgcaaaaagctaacccctgctcagcagggaaaaatggctgtaaaaaggctttttttattta
